# Metabolic reprogramming via SIRT2-deficient microglial large extracellular vesicles ameliorates alzheimer’s pathology

**DOI:** 10.1186/s12974-026-03956-3

**Published:** 2026-07-17

**Authors:** Xiaolu Tang, Rui Chen, Jiawei Xing, Qing Huang, Lv Luo, Chaoyue Ouyang, Fubin Ma, Jie Wen, Jiahao Zhang, Lingyun Kong, Yujie Cai, Yan Wang, Lili Cui

**Affiliations:** 1 Guangdong Key Laboratory of Age-Related Cardiac and Cerebral Diseases, Affiliated Hospital of Guangdong Medical University, South Renmin road 57, Zhanjiang, Guangdong 524000 China; 2 The Marine Biomedical Research Institute of Guangdong, School of Ocean and Tropical Medicine, Guangdong Medical University, Zhanjiang, Guangdong 524000 China; 3 Key Laboratory of Prevention and Treatment of Cardiovascular and Cerebrovascular Diseases of Ministry of Education, Ganzhou Key Laboratory of Neuroinflammation Research, School of Basic Medicine Sciences, Gannan Medical University, Ganzhou, 341000 China; 4 State Key Laboratory of Mechanism and Quality of Chinese Medicine, Faculty of Chinese Medicine, Macau University of Science and Technology, Taipa, Macau SAR 999078 China; 5 Department of Neurology, Xiangyang Hospital of Traditional Chinese Medicine, Xiangyang, Hubei 441000 China

**Keywords:** Alzheimer’s disease (AD), Oxidative phosphorylation, Glycolysis, Extracellular vesicles (EVs), SIRT2

## Abstract

**Background:**

Current therapies for Alzheimer’s disease (AD) offer only symptomatic relief, highlighting the urgent need for disease-modifying approaches capable of halting or reversing neurodegeneration. Extracellular vesicles (EVs) have attracted growing interest as therapeutic vehicles owing to their inherent capacity to bypass the blood-brain barrier and deliver complex biological cargo to the central nervous system.

**Methods:**

Here, we examined whether large EVs (LEVs) derived from microglia with stable Sirtuin-2 knockdown (SIRT2-KD) confer the neuroprotective effects associated with SIRT2 inhibition. LEVs harvested from SIRT2-KD microglia were administered intranasally to APP/PS1 mice. We assessed microglial uptake of LEVs, along with subsequent changes in cellular metabolism, migration toward amyloid-beta (Aβ) plaques, phagocytic activity, and downstream pathological and behavioral outcomes. Proteomic and acetylomic profiling were employed to characterize the molecular cargo of LEVs-SIRT2-KD.

**Results:**

LEVs-SIRT2-KD were readily internalized by microglia in vivo following intranasal delivery. Uptake of these vesicles markedly enhanced microglial bioenergetics, driving coordinated upregulation of both oxidative phosphorylation and glycolysis. This metabolic shift was accompanied by improved microglial recruitment to Aβ plaques and increased phagocytic clearance. Consequently, treated mice showed reduced Aβ plaque deposition, restored synaptic integrity, and reversal of cognitive deficits. Proteomic and acetylomic analyses revealed that LEVs-SIRT2-KD are selectively enriched in proteins and acetylation modifications linked to energy metabolism and phagocytic function, offering a mechanistic basis for the observed metabolic reprogramming.

**Conclusion:**

Together, these results identify LEVs as a critical vesicle subtype mediating the effects of SIRT2 knockdown and support a cell-free therapeutic strategy for AD centered on EVs-driven metabolic reprogramming of microglia.

**Graphical Abstract:**

**Figure:**
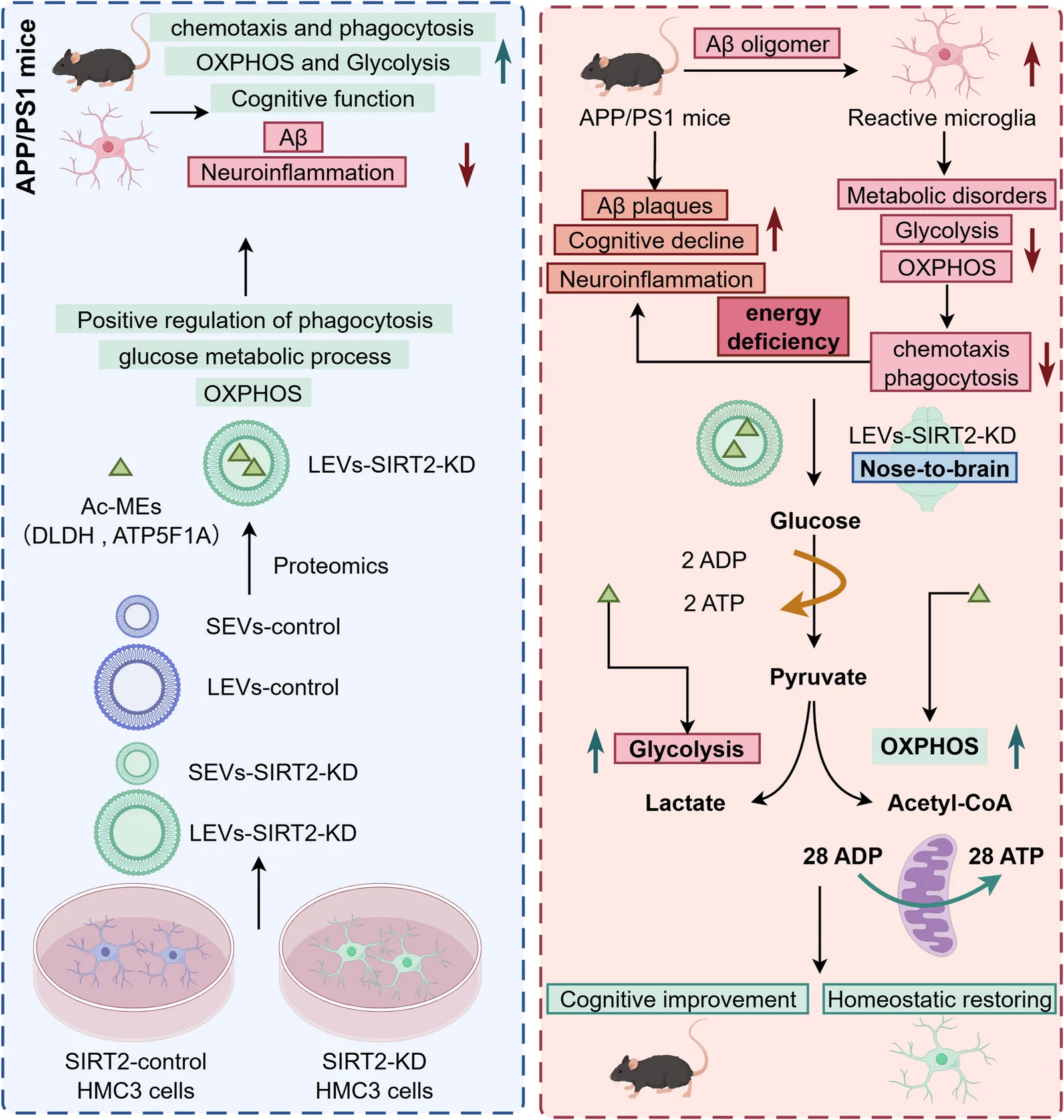
In chronic Alzheimer’s disease (AD), microglial metabolism is severely compromised. We demonstrate that SIRT2-KD-derived LEVs rescue this collapse by delivering a specific cargo profile. Notably, this profile is enriched with acetylated metabolic enzymes (Ac-MEs), such as DLDH and ATP5F1A. This transfer drives metabolic reprogramming and restores bioenergetics, empowering microglia to enhance chemotaxis and Aβ clearance. These findings reveal a novel precision nanotherapy strategy involving the delivery of functionally modified enzymes.

**Supplementary Information:**

The online version contains supplementary material available at https://doi.org/10.1186/s12974-026-03956-3.

## Background

Alzheimer’s disease (AD), the most prevalent neurodegenerative disorder and the leading cause of dementia, is characterized by progressive cognitive decline that poses a significant burden on global public health [1]. Accumulating evidence supports that the abnormal aggregation and deposition of amyloid-beta (Aβ) peptides in the brain serve as both an initiating event and a fundamental pathological hallmark of AD [2]. The imbalance between Aβ production and clearance leads to the formation of insoluble plaques, triggering a cascade of downstream pathologies, including oxidative stress, chronic neuroinflammation, synaptic dysfunction, and neuronal death [3–5]. Consequently, enhancing Aβ clearance has emerged as a crucial therapeutic strategy. Although anti-Aβ monoclonal antibodies, such as Lecanemab [6] and Donanemab [7], have shown efficacy in plaque removal, their cognitive benefits are modest and are often accompanied by adverse effects like amyloid-related imaging abnormalities (ARIA) [8–11]. This underscores the urgent need for novel, effective, and safer approaches to promote Aβ clearance.

Microglia, the brain’s resident innate immune cells, constitute the first line of defense against Aβ accumulation [12]. Recent advances highlight that targeted modulation of microglial function can significantly alleviate AD pathology. For instance, intranasal administration of the immunomodulator protollin activates microglia, facilitating Aβ clearance and improving memory in AD models [13]. Similarly, gamma-frequency sensory stimulation promotes microglial recruitment to plaques, reducing Aβ burden [14]. Notably, the natural compound sodium rutin enhances microglial phagocytosis by improving mitochondrial energy metabolism, thereby mitigating AD pathology [15]. These studies converge on the concept that enhancing both the chemotactic migration of microglia toward Aβ plaques and their subsequent phagocytic clearance represents a highly promising intervention point in AD progression.

Microglial migration and phagocytosis are highly energy‑demanding processes that require coordinated regulation of both glycolysis and oxidative phosphorylation [16, 17, 52]. Emerging evidence indicates that metabolic dysfunction is a hallmark of AD microglia, characterized by impaired glucose metabolism and mitochondrial dysfunction [18]. Such metabolic deficits directly compromise the ability of microglia to migrate toward Aβ plaques and clear them efficiently [18]. Thus, restoring microglial energy metabolism may serve as a viable therapeutic target for enhancing Aβ clearance in AD.

Sirtuin 2 (SIRT2), a NAD⁺-dependent deacetylase highly expressed in the central nervous system, plays a pivotal role in regulating metabolism, inflammation, and aging [19, 20]. A strong correlation exists between SIRT2 and AD; its expression is elevated in AD patients and mouse models, and genetic polymorphisms in SIRT2 are linked to increased AD susceptibility [21–23]. Critically, inhibition or genetic knockout of SIRT2 has been shown to improve cognitive function and alleviate Aβ pathology in AD mouse models [22, 24–26]. Mechanistically, SIRT2 silencing in microglia promotes a protective M2-like phenotype, suppresses excessive neuroinflammation, and enhances their reparative functions [27–32]. These findings establish SIRT2 as a promising therapeutic target for modulating the cerebral immune microenvironment in AD.

However, the clinical translation of SIRT2-targeted therapies faces substantial hurdles. Small molecule SIRT2 inhibitors suffer from poor blood-brain barrier (BBB) permeability, significant off-target effects, and short half-lives, with some studies even reporting exacerbated peripheral inflammation [33]. Conversely, viral vector-mediated gene therapy carries risks of high immunogenicity and insertional mutagenesis [34]. This therapeutic gap highlights the need for a strategy that can safely and efficiently deliver the beneficial effects of SIRT2 modulation—specifically within microglia—to the brain.

Extracellular vesicles (EVs), nanoscale lipid bilayer particles actively secreted by cells, have emerged as powerful intercellular communication mediators and therapeutic agents [35, 36]. Based on their biogenesis, EVs are broadly classified into large EVs (LEVs; 50–1000 nm), which bud directly from the plasma membrane, and small EVs (SEVs; 40–160 nm), originating from multivesicular bodies [37–39]. EVs carry a rich cargo of proteins, nucleic acids, and lipids from their parent cells, offering excellent biocompatibility, low immunogenicity, and the ability to bypass biological barriers, including the BBB via non-invasive routes like intranasal administration [40–43]. Notably, microglia-derived EVs hold particular promise. SEVs from anti-inflammatory M2 microglia have been shown to alleviate Aβ pathology and improve cognition [44], while the protein cargo of LEVs can directly inhibit Aβ aggregation [37]. These findings position microglial EVs as ideal vectors to deliver complex therapeutic signals—such as those induced by SIRT2 knockdown—directly to diseased brain regions.

In this study, we leveraged this concept by developing a stable SIRT2-knockdown human microglial cell line (HMC3) to investigate whether the therapeutic benefits of SIRT2 silencing could be transferred via its secreted EVs. Given the distinct biogenesis and cargo profiles of LEVs and SEVs, we isolated both subtypes to conduct a comparative analysis of their therapeutic potential in APP/PS1 mouse models. We then systematically evaluated the effects of these EVs on AD-related pathology and cognitive function, and explored the underlying mechanisms through a combination of behavioral assessments, histopathological analyses, cellular energy metabolism measurements, and multi-omics profiling (proteomics and acetylomics). Our results demonstrate that intranasally administered LEVs derived from SIRT2-KD microglia (LEVs-SIRT2-KD) significantly alleviate Aβ plaque deposition, restore synaptic plasticity, and rescue cognitive deficits in APP/PS1 mice. Mechanistically, we found that LEVs-SIRT2-KD concurrently enhance oxidative phosphorylation and glycolysis in microglia, reversing Aβ-induced metabolic dysfunction and thereby improving microglial migration and phagocytic clearance of Aβ plaques. Proteomic and acetylomic analyses further revealed that LEVs-SIRT2-KD carry a unique cargo: they inherit the intrinsic energy-metabolism predisposition of LEVs, acquire additional stress-resilience and catabolic machinery upon SIRT2 knockdown, and harbor specific acetylation marks on key metabolic enzymes—including DLDH, MDH2, ATP5F1A, ALDOA, and SHMT—that collectively position them to reprogram recipient microglial energy metabolism. Together, our findings identify LEVs as the key functional vesicle subtype mediating the benefits of SIRT2 knockdown and establish a mechanistic link between SIRT2-regulated metabolic reprogramming and microglial immune function, offering a novel, cell-free therapeutic paradigm for AD.

## Methods

### Construction of SIRT2-knockdown HMC3 stable cell culture

HMC3 cells were cultured in 6‑cm dishes in minimum essential medium (MEM) supplemented with 10% fetal bovine serum and 1% penicillin/streptomycin until they reached 40%–50% confluence, which is optimal for lentiviral infection. Lentiviral vectors encoding shRNA‑control or shRNA‑SIRT2 (VectorBuilder, China) were thawed, and the volume needed to achieve a multiplicity of infection of 25 was calculated and diluted with MEM. Meanwhile, MEM containing polybrene was prepared, and 1.8 mL of this solution was added to each dish after removal of the original medium. Subsequently, 200 µL of the diluted lentivirus was evenly distributed into each dish to initiate infection. After 24 h, the medium was replaced with fresh MEM. At 72 h post‑infection, cells were selected with 1.5 µg/mL puromycin (P8230, Solarbio, China) to establish stable expression lines, and this puromycin concentration was maintained during subsequent selection. Stable cell lines were obtained after three consecutive rounds of selection and were then harvested for further analysis.

### Isolation of EVs from HMC3 microglia cells

SIRT2‑control and SIRT2‑KD HMC3 cells were cultured in 15‑cm dishes until they reached 80% confluence. The culture medium was then replaced with exosome‑free MEM, and the cells were incubated for another 24 h. After incubation, the conditioned medium was collected and stored at − 80 °C until use.

For EV isolation, the frozen medium was thawed and centrifuged at 500 × g for 10 min at 4 °C to remove cell debris. The supernatant was retained and centrifuged again at 4,000 × g for 10 min at 4 °C, and the resulting pellet was discarded. The supernatant was then subjected to centrifugation at 16,000 × g for 30 min at 4 °C. The pellet was collected, resuspended in sterile PBS, and centrifuged again under the same conditions (16,000 × g, 30 min, 4 °C). After this second spin, the pellet was resuspended in a small volume of sterile PBS to obtain the large EVs (LEVs) fraction.

The supernatant from the first 16,000 × g centrifugation step was filtered through a 0.45‑µm membrane and transferred to a 70‑mL ultracentrifuge tube. After balancing the tubes to within 0.01 g, the supernatant was ultracentrifuged at 100,000 × g for 120 min at 4 °C. The resulting pellet was resuspended in sterile PBS, diluted, and subjected to a second ultracentrifugation step under the same conditions (100,000 × g, 120 min, 4 °C). The final pellet was resuspended in a minimal volume of sterile PBS to obtain the small EVs (SEVs) fraction.

### EVs labeled by DiI

To prepare the stock solution, 10 mg of DiI (DiIC18(3), 4072ES101, YESEN, China) was dissolved in 4 mL of dimethyl sulfoxide (DMSO), yielding a final concentration of 2.5 mg/mL (2.677 mol/L). The stock solution was stored at − 20 °C. For EVs labeling, the DiI stock solution was added to the collected HMC3 culture medium to a final concentration of 5 µM, and the mixture was incubated at 37 °C for 20 min. DiI‑labeled LEVs and SEVs were then isolated following the same EVs extraction protocol described above [45].

### Characterization of SEVs and LEVs derived from HMC3 cells

SEVs and LEVs isolated from SIRT2‑control and SIRT2‑KD HMC3 cells were designated as LEVs‑control, SEVs‑control, LEVs‑SIRT2‑KD, and SEVs‑SIRT2‑KD, respectively. Particle size distribution was determined by nanoparticle tracking analysis (NTA), and morphology was examined by transmission electron microscopy (TEM). Protein concentration was measured using the BCA assay. In addition, the presence of EVs marker proteins—including CD63, Alix, flotillin‑2, and ARF6—was assessed by Western blot.

### Protein extraction from EVs

EVs were lysed in an equal volume of RIPA lysis buffer (P0013C, Beyotime, China) supplemented with 1 mM PMSF and a protease inhibitor cocktail (1×, 04693116001, Roche, Switzerland). The lysates were incubated on ice for 30 min and then centrifuged at 14,000 × g for 10 min to collect the protein supernatant. Protein concentration was determined using the BCA assay.

### TEM analysis of EVs morphology

For TEM analysis, 10 µL of the EVs sample was placed onto a 300‑mesh copper grid and allowed to adhere for 2 min. Excess liquid was then carefully removed from the edge of the grid with filter paper. The sample was stained with 10 µL of 0.5% (w/v) uranyl acetate for 2 min, after which the excess stain was removed with filter paper. The grid was then examined under a transmission electron microscope for morphological assessment.

### Animals

Seven-month-old male APPswe/PSEN1dE9 (APP/PS1) transgenic mice (B6C3H) and their wild-type littermates were obtained from Hangzhou Ziyuan Experimental Animal Technology Co., Ltd. (qualified permit number: 20240424Abbz0105000619). The mice were housed in a specific pathogen-free (SPF) animal facility at Guangdong Medical University under a 12-h light/dark cycle with free access to food and water. All experimental procedures complied with the regulations established by the Institute of Neurology at Guangdong Medical University and were approved by the Ethical Review Committee of Guangdong Medical University (approval number: GDMU-2024-000030).

### Behavioral experiments

Eight‑month‑old male APP/PS1 mice and their wild‑type littermates were randomly assigned to six experimental groups: wild‑type, APP/PS1, SEVs‑control, LEVs‑control, SEVs‑SIRT2‑KD, and LEVs‑SIRT2‑KD. Mice in each group received intranasal administration of the corresponding PBS, SEVs, or LEVs using a 10‑µL pipette at a dose of 0.5 × 10⁹ particles per administration (approximately 10 µg) every other day. After 30 consecutive administrations, when the mice reached 10 months of age, behavioral tests were performed.

### The nest-building test

This is a significant approach for evaluating the self-care abilities, emotional states, and social behaviors of mice [46, 47]. The mice were transferred to individual cages approximately one hour before the start of the dark cycle. Each cage was furnished with nesting materials consisting of eight pieces (5 × 5 cm) of white paper. Changes in the nesting materials within the test cages were recorded by photography over 7 days. Every morning at 8:30, the nesting behavior of the mice was comprehensively evaluated on a scale of 1 to 5. The scoring criteria were as follows: (a) A score of 1 was assigned when the nesting material remained largely unaltered, with over 90% remaining undamaged. (b) A score of 2 was assigned when the material was partially shredded, retaining 50%–90% of its original form. (c) A score of 3 was allotted when the majority (50%–90%) of the material was shredded, no distinct nest was formed, and the shredded material was dispersed throughout the cage. (d) A score of 4 was granted when > 90% of the material was shredded and gathered in a quarter of the cage to form a flat nest. (e) A score of 5 was awarded when > 90% of the material was shredded and arranged into an almost perfect cup-shaped nest, with > 50% of the nest wall exceeding the height of the mouse. The cumulative scores for each group of mice over the 7 days were analyzed to evaluate changes in cognitive behavior following drug administration.

### The Y‑maze test

The Y‑maze test was used to assess spontaneous alternation behavior, reflecting learning, memory, and spatial navigation in mice [48]. The apparatus consisted of three arms of equal size (each approximately 10 cm wide and 30 cm long) arranged in a Y‑shape with an inter‑arm angle of 120°. Before testing, mice were acclimated to the environment for 1 h. At the start of the test, each mouse was placed at the central intersection and allowed to explore freely for 5 min. A video‑tracking system (Animal Behavioral Analyze System, JLBehv, Shanghai Jiliang Software Technology, China) with an overhead camera recorded the movements for subsequent analysis. The spontaneous alternation index was calculated as (number of spontaneous alternations / (total arm entries − 2)) × 100% and used as an indicator of short‑term and spatial memory.

### The Morris water maze test

The Morris water maze test was used to evaluate spatial learning and memory in rodents, with a particular focus on hippocampal function [48]. The apparatus consisted of a circular pool (120 cm in diameter) and a circular platform (10 cm in diameter). Water temperature was maintained at approximately 22–25 °C. Curtains with four distinct geometric patterns surrounded the pool. A camera mounted above the center of the pool was connected to a video‑tracking system (Morris Maze, Zhongshi Technology, China) to record the movements of the rodents throughout the experiment.

The experimental procedure consisted of three phases: visible platform, hidden platform, and probe test. In the visible platform phase, the platform was placed 1.5 cm above the water surface, and mice were introduced into the quadrant opposite the platform, facing the pool wall. A camera and tracking system recorded swimming speed. During the hidden platform phase, the water was made opaque with non‑toxic titanium dioxide, and the platform was submerged 1 cm below the surface. In each trial, mice were randomly placed into one of the four quadrants and given 90 s to find the hidden platform. If a mouse failed to locate it within this time, it was guided to the platform and allowed to stay there for 10 s. The latency to reach the platform was recorded. After five days of training, the platform was removed, and mice were allowed to swim freely for 90 s. The time spent in the target quadrant (where the platform had been located) and the number of crossings over the former platform position were recorded as measures of spatial memory.

### Tissue acquisition and sample preparation

Mice were deeply anesthetized with tribromoethanol (200 µL per 10 g body weight) and then perfused transcardially with PBS (pH 7.4). Once the liver appeared blanched, the brain was rapidly removed. The left and right hemispheres were separated by cutting along the sagittal suture. The left hemisphere was dissected to isolate the hippocampus and cortex, which were immediately frozen and stored at − 80 °C for subsequent analyses. The right hemisphere was fixed in 4% paraformaldehyde (PFA) at 4 °C. After 48 h of fixation, the right hemisphere was dehydrated through 20% and 30% sucrose in PBS, then sectioned into 30‑µm‑thick coronal or sagittal slices using a cryostat (HM525 NX; Thermo Scientific, USA).

### Protein extraction

For protein extraction from mouse hippocampal tissue, 100 µL of RIPA lysis buffer (P0013C, Beyotime, China) supplemented with 1 mM phenylmethylsulfonyl fluoride (PMSF; ST506, Beyotime, China) and a 1× protease inhibitor cocktail (04693116001, Roche, Switzerland) was added to an Eppendorf tube containing the tissue sample. The tissue was carefully minced into small pieces using ophthalmic scissors, followed by the addition of another 300 µL of lysis buffer. The mixture was incubated on ice for 30 min. After incubation, the samples were sonicated in an ice bath using an ultrasonic processor (XM-26 A-30, Kunshan Ultrasonic Instrument Co., China) set to 300 W with 5‑second pulses and 15‑second intervals for a total of 15 cycles (4 min). The homogenate was then centrifuged at 14,000 × g for 10 min at 4 °C to collect the protein supernatant. Protein concentration was determined using the bicinchoninic acid (BCA) assay (23225, Thermo Scientific, USA).

### Western blot

Protein samples were separated by 5%–15% SDS‑PAGE and then transferred onto polyvinylidene fluoride (PVDF) membranes (IPVH00010, Millipore, USA). The membranes were blocked with 5% non‑fat milk at room temperature for 1 h to prevent non‑specific binding, followed by overnight incubation with primary antibodies at 4 °C. The following primary antibodies were used: rabbit anti-CD86(1:1000, SJ20-00, HUABIO, China), rabbit anti-CD206 (1:1000, ab64693, Abcam, UK), rabbit anti‑CD63 (1:1000, 67605‑1‑Ig, Proteintech, China), rabbit anti‑Alix (1:1000, 12422‑1‑Ap, Proteintech, China), rabbit anti‑flotillin‑2 (1:1000, 28208‑1‑Ig, Proteintech, China), rabbit anti‑ARF6 (1:1000, 20225‑1‑AP, Proteintech, China), rabbit anti‑Iba1 (1:1000, 016‑20001, WAKO, Japan), rabbit anti‑SIRT2 (1:1000, 19655‑1‑Ig, Proteintech, China), mouse anti‑Aβ (1:1000, clone 6E10, 803001, BioLegend, USA), and mouse anti‑APP (1:1000, 60342‑1‑Ig, Proteintech, China). After washing, the membranes were incubated with horseradish peroxidase (HRP)‑conjugated secondary antibodies: goat anti‑rabbit (1:5000, A21020, Abbkine, China) and goat anti‑mouse (1:1000, RGAM001, Proteintech, China). Protein signals were visualized using a chemiluminescence detection kit (P90720, Millipore, USA), and band intensities were quantified with ImageJ software.

### Immunofluorescence

For immunofluorescence analysis, brain sections (coronal or sagittal) or fixed cells were blocked in PBS containing 3% bovine serum albumin (BSA) and 0.5% Triton X-100 for 1 h at room temperature. Samples were then incubated with primary antibodies overnight at 4 °C. The primary antibodies used were as follows: goat anti‑Iba1 (1:300, ab5076, Abcam), rabbit anti‑Iba1 (1:200, 016‑20001, Wako), mouse anti‑Aβ (1:1000, clone 6E10, 803001, BioLegend), rabbit anti‑GFAP (1:1000, ab7260, Abcam), rabbit anti‑CD63 (1:500, 67605‑1‑Ig, Proteintech, China), rabbit anti‑SYN (1:100, 17785‑1‑AP, Proteintech), rat anti‑PSD95 (1:250, HA601406, HUABIO), rabbit anti‑CD68 (1:500, HA722737, HUABIO), mouse anti‑CD86 (1:100, PTM‑5334, PTM‑BioLab), and mouse anti‑CD206 (1:100, PTM‑6020, PTM‑BioLab).

The next day, samples were allowed to warm to room temperature for at least 30 min, then washed three times with PBS. Secondary antibodies were then added, and the samples were incubated for 1 h at room temperature in the dark. The following fluorescent secondary antibodies were used: Alexa Fluor^®^ 555 goat anti‑mouse IgG H&L (1:500, ab150118, Abcam), Alexa Fluor^®^ 488 goat anti‑rat IgG H&L (1:500, ab150157, Abcam), Alexa Fluor^®^ 555 goat anti‑rabbit IgG H&L (1:500, ab150078, Abcam), Alexa Fluor^®^ 488 goat anti‑rabbit IgG H&L (1:500, ab150077, Abcam), Alexa Fluor^®^ 488 goat anti‑mouse IgG (H + L) (1:500, A11029, Thermo Fisher), and Alexa Fluor^®^ 647 donkey anti‑rabbit IgG H&L (1:500, ab150075, Abcam).

After incubation, samples were washed three times with PBS. Brain sections were then mounted onto glass slides, air‑dried at 50 °C, and coverslipped with an anti‑fade mounting medium containing DAPI for nuclear staining. Images were acquired using a confocal microscope (FV3000, Olympus, Japan) or a digital slide scanner (Imager.Z2, Zeiss, Germany).

### Aβ_42_ oligomers preparation

Human Aβ_1‑42_ (Aβ_42_) peptides, as well as fluorescein amidite‑labeled (FAM‑Aβ_42_) and cyanine 5‑labeled (Cy5‑Aβ_42_) Aβ_42_ peptides, were synthesized by QYAOBIO (Shanghai, China). To prepare the peptide solution, 222 µL of 1,1,1,3,3,3‑hexafluoro‑2‑propanol (HFP, 52517, Sigma) was added to 1 mg of Aβ_42_ peptide, yielding a final concentration of 1 mM. The mixture was incubated at room temperature for 60 min, then placed on ice for 10 min. The solution was aliquoted into two tubes (111 µL per tube), and the caps were left open. The tubes were then incubated overnight at room temperature in a fume hood, followed by drying in a vacuum concentrator for 10 min to form a thin, transparent Aβ_42_ film on the tube walls. To prepare a 5 mM stock solution, 0.5 mg of the peptide was dissolved in 22.2 µL of freshly prepared anhydrous DMSO. This stock was then diluted to a final concentration of 100 µM with MEM and incubated at 4 °C for 24 h to allow the formation of Aβ_42_ oligomers.

### Scratch assay

Black straight lines were drawn on the bottom of a 24‑well plate as reference marks. HMC3 cells were seeded into the wells and cultured until they reached 95% confluence. A vertical scratch was then made across each well using a 200‑µL pipette tip. The plate was gently rinsed with PBS to remove detached cells, and Aβ_42_ oligomers (10 µM) were added to the designated treatment groups. After 3 h of incubation, LEVs‑SIRT2‑KD (2 µg/mL), 2‑deoxy‑D‑glucose (2‑DG; 30 mM, HY‑13966, MCE), or rotenone/antimycin A (Rot/AA; 0.25 µM, 103015‑100‑1, Agilent) were administered to the respective groups. Images of the scratched area were captured at 0, 24, and 48 h post‑scratch using a microscope with a 10× objective. Migration distance and the percentage of the closed area were calculated to assess the effect of LEVs‑SIRT2‑KD on HMC3 cell migration.

### Phagocytosis assay

#### Immunofluorescence

HMC3 cells were seeded in confocal microplates (15‑mm diameter) and cultured at 37 °C in a 5% CO₂ atmosphere. Upon reaching 40%–50% confluence, the cells were treated with Aβ_42_ oligomers (10 µM) in designated wells. After 3 h, the cells were co‑treated with either LEVs‑SIRT2‑KD (2 µg/mL) or PBS, along with Cy5‑ or FAM‑labeled Aβ_42_ oligomers (1 µM). Following a 21‑hour incubation, the cells were fixed with 4% PFA in PBS. Internalization of the labeled Aβ_42_ was then assessed by immunofluorescence staining for α‑tubulin, with nuclei counterstained by DAPI.

#### Flow cytometry analysis

HMC3 cells were seeded in 6‑ or 12‑well plates and cultured overnight. When the cells reached approximately 50% confluence, Aβ_42_ oligomers (10 µM) were added to the designated wells. After 3 h of incubation, the cells were treated with LEVs‑SIRT2‑KD (2 µg/mL), 2‑DG (30 mM, HY‑13966, MCE, China), Rot/AA (0.25 µM, 103015‑100‑1, Agilent, USA), or Cytochalasin D (CytoD, 20 mM, A574218, Ambeed, China) according to the experimental groups. Following an additional 2‑hour incubation, FAM‑labeled Aβ_42_ oligomers (1 µM) were added, and the culture was continued for another 21 h.

The cells were then harvested, digested with trypsin (0.25%; Gibco, USA), and analyzed by flow cytometry using the FAM channel. Forward scatter (FSC) and side scatter (SSC) were used to gate viable cells. The fluorescence signal from CytoD‑treated cells was used as a background reference to distinguish non‑specifically bound FAM‑Aβ_42_ from internalized FAM‑Aβ_42_, allowing identification of cells that had actively phagocytosed the labeled Aβ_42_.

#### Seahorse XFe cell energy metabolism phenotype assay

HMC3 cells were seeded in a 24‑well plate (102340‑100‑1, Agilent, USA) and incubated overnight. After 6 h of serum starvation in MEM basal medium, Aβ_42_ oligomers (10 µM) were added to the designated treatment groups (Aβ_42_ alone and LEVs‑SIRT2‑KD + Aβ_42_). Following a 3‑hour incubation, LEVs‑SIRT2‑KD (2 µg/mL) was added to the relevant groups (LEVs‑SIRT2‑KD alone and LEVs‑SIRT2‑KD + Aβ_42_) for 21 h. Oxygen consumption rate (OCR) and extracellular acidification rate (ECAR) were then measured using a Seahorse XFe24 Cell Energy Metabolism Analyzer (Agilent Technologies, USA), following the manufacturer’s protocols for the Cell Mitochondrial Stress Test Kit (103015‑100‑1, Agilent) and the Glycolysis Rate Test Kit (103344‑100‑1, Agilent).

#### ATP detection assay

HMC3 cells were seeded in 6‑well plates and cultured overnight. The cells were then starved in MEM basal medium for 6 h, during which they reached approximately 50% confluence. Aβ_42_ oligomers (10 µM) were added to the designated groups (Aβ_42_ alone and LEVs‑SIRT2‑KD + Aβ_42_). After an additional 3 h, LEVs‑SIRT2‑KD (2 µg/mL) was added to the relevant groups (LEVs‑SIRT2‑KD alone and LEVs‑SIRT2‑KD + Aβ_42_), and the cells were incubated for another 21 h. ATP levels were then measured using the Enhanced ATP Detection Kit (S0027, Beyotime, China) according to the manufacturer’s protocol.

#### Quantitative proteomics

A quantitative proteomic analysis of the vesicle proteins was performed at Zhongke Xinlife Biotechnology Co., Ltd. (APTBIO, Shanghai, China). The experimental procedure was as follows: SEVs and LEVs samples were lysed using SDT lysis buffer, which contained 4% SDS and 100 mM Tris-HCl (pH 7.6), for protein extraction. Protein quantification was performed using the BCA assay. An aliquot of 15 µg from each protein sample was denatured with 5× loading buffer and subjected to SDS-PAGE using a 4%–20% gradient gel under a constant voltage of 180 V for 45 min. Subsequently, the gels were stained with Coomassie Brilliant Blue R-250 to assess the sample quality. Equal quantities of each sample were combined to prepare pooled quality control (QC) samples.

All samples, including QC samples, were subjected to trypsin digestion using the filter-aided sample preparation (FASP) method. The resulting enzymatic peptides were desalted using a C18 cartridge, lyophilized, and reconstituted in 40 µL of 0.1% formic acid. The peptide concentration was determined by measuring the absorbance at 280 nm. Standard peptides with an internal retention time (iRT) were incorporated into each sample.

Peptides were separated using an upgraded high-performance liquid chromatography (HPLC) system (Vanquish Neo, Thermo Scientific, USA) coupled with an Astral high-resolution mass spectrometer, using data-independent acquisition (DIA) mass spectrometry. The parameters for mass spectrometry detection were as follows: a positive ion mode; a mass-to-charge ratio (m/z) scanning range of 380–980 for MS1, a resolution of 240,000 at 200 m/z, a normalized automatic gain control (AGC) target of 500%, and a maximum injection time (IT) of 5 ms. The MS2 analysis used the DIA mode, with 299 scanning windows, an isolation window of 2 m/z, a higher-energy collisional dissociation (HCD) collision energy of 25 electron volts (eV), a normalized AGC target of 500%, and a maximum IT of 3 ms.

DIA data were processed using DIA-NN software with the following parameters: trypsin as the protease, a maximum of one missed cleavage site, carbamidomethylation of cysteine (C) as a fixed modification, oxidation of methionine (M), and acetylation of the protein N-terminus as dynamic modifications. The false discovery rate (FDR) for protein identification was < 1%. The criteria for screening differentially expressed proteins (DEPs) were an expression fold change > 1.5 and *P* < 0.05. Finally, the bioinformatics tools Blast2GO and KAAS were used to perform gene ontology (GO) functional annotation and Kyoto encyclopedia of genes and genomes (KEGG) pathway enrichment analysis on the set of DEPs.

#### Acetylation proteomics

Following protein extraction, 15 µg of EV protein was subjected to SDS-PAGE to assess sample quality and consistency. Subsequently, trypsin was used to perform in-solution digestion. To achieve complete reduction and alkylation, the samples were sequentially treated with dithiothreitol (DTT) at a final concentration of 10 mM at 37 °C for 2 h, followed by iodoacetamide (IAA) at a final concentration of 50 mM at room temperature in the dark for 1 h. The samples were then diluted with 6× volumes of 100 mM Tris buffer, trypsin was added at an enzyme-to-protein ratio of 1:25 (w/w), and digestion was allowed to proceed overnight at 37 °C for 15–18 h. The pH was adjusted to approximately 3 by acidification with 20% trifluoroacetic acid (TFA) at a 1:20 volume ratio, which halted the digestion. The resulting digested peptides were desalted using a C18 desalting column, freeze-dried, and subsequently enriched for acetylated peptides using an acetylated antibody enrichment kit (PTMScan^®^ Acetyl-Lysine Motif [Ac-K] Kit, CST #13416). The vacuum-concentrated enriched peptides were resuspended in 0.1% formic acid. A total of 2 µg of these peptides was combined with iRT standard peptides for subsequent DIA mass spectrometry. Mass spectrometry analysis was performed using a nanoflow liquid chromatography system (Vanquish Neo, Thermo Scientific) for peptide separation, coupled with an Astral high-resolution mass spectrometer for DIA. The mass spectrometry parameters were set as follows: positive ion mode, MS1 scan range of 380–980 m/z, resolution of 240,000 (@200 m/z), normalized AGC target of 500%, and maximum IT of 5 ms. MS2 was operated in DIA mode with 300 scan windows, an isolation window of 2 m/z, HCD collision energy of 25 eV, normalized AGC target of 300%, and maximum IT of 3 ms. The acquired DIA data were processed using Spectronaut software with the following parameters: enzyme trypsin, a maximum of two missed cleavage sites, fixed modification of C, and variable modifications, including Oxidation (M), Acetylation (Protein N-term), and Acetylation (K). Protein identification was achieved with an FDR of < 1%. The criteria for selecting differentially acetylated modified peptides were a fold change > 1.5 and *P* < 0.05. Subsequently, Blast2GO and KAAS were used to perform GO functional annotation and KEGG pathway enrichment analysis of the set of differentially acetylated peptides.

#### Hippocampal transcriptomes by RNA sequencing

Total RNA was extracted from the hippocampal tissues of each mouse group using TRIzol reagent (Invitrogen, USA). RNA integrity was confirmed through a quality evaluation. A total of 1 µg RNA was used to construct sequencing libraries using the NEBNext Ultra RNA Library Prep Kit (New England Biolabs, USA). The quality of these libraries was assessed using an Agilent 2100 Bioanalyzer (Agilent Technologies, USA) and found satisfactory. Subsequently, paired-end sequencing was performed using the Illumina NovaSeq 6000 platform (Illumina, USA). The raw sequencing data were subjected to QC processes, during which adapter sequences were excised, and low-quality reads (those containing more than five ambiguous bases, Ns) were removed. The resultant high-quality data were aligned to the mouse reference genome using HISAT2 software, and the gene expression levels in each sample were quantified using FeatureCounts and presented as fragments per kilobase of transcript per million mapped reads. Differential gene expression analysis was performed using DESeq2 software, with significance criteria set at |log2 fold change| > 1 and *P* < 0.05. Furthermore, the multiGSEA software package was used to perform gene set enrichment analysis (GSEA), based on the Reactome database for predefined gene sets, with a significance threshold of |normalized enrichment score| > 1 and *P* < 0.05.

### Statistical analysis

All behavioral assessments and image analyses were conducted by investigators blinded to group allocation. Data are presented as mean ± standard error of the mean (SEM). For comparisons involving two groups, two-tailed Student’s t-tests were applied. For comparisons involving three or more groups, one‑way or two‑way analysis of variance (ANOVA) was used as appropriate. Following ANOVA, post‑hoc comparisons were conducted using Holm‑Šidák, Dunnett’s T3, or Tukey’s tests, depending on the variance structure and the specific hypotheses tested (e.g., comparisons with a control group or pairwise comparisons among all groups). All statistical analyses were carried out using GraphPad Prism, and statistical significance was set at *P* < 0.05.

## Results

### Characterization of EVs derived from SIRT2-KD HMC3 cells

**Fig. 1:**
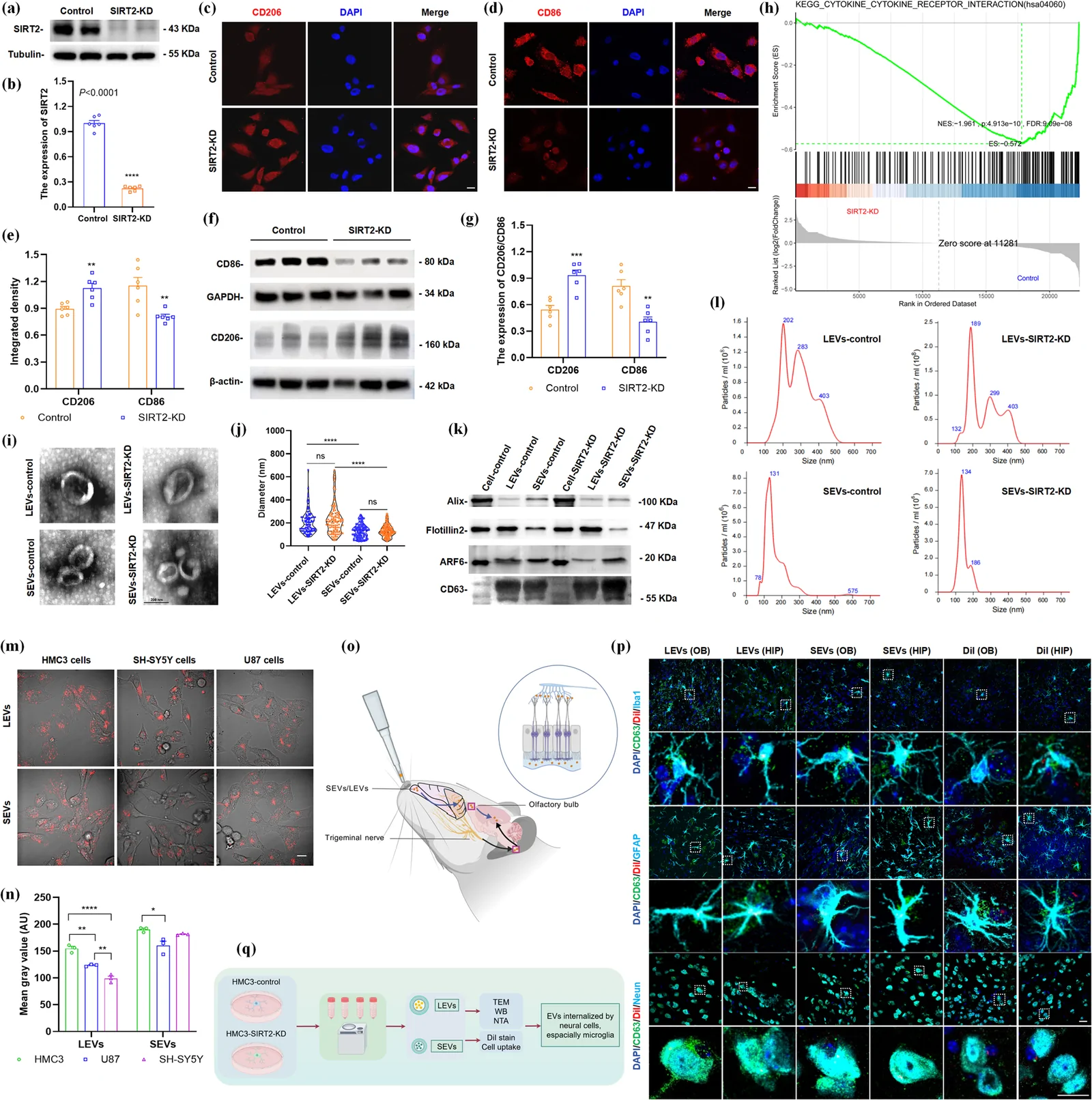
Characterization of large and small extracellular vesicles derived from HMC3 microglial cells. **a** Western blot analysis of SIRT2 protein expression in HMC3 cells following lentiviral transfection. **b** Densitometric quantification of SIRT2 expression levels (*n* = 6). **c-d** Immunofluorescence analysis of CD206 and CD86 in SIRT2-knockdown HMC3 cells. **e** Quantification of CD206 and CD86 fluorescence intensity in HMC3 cells (*n* = 6). **f** Western blot analysis of CD206 and CD86 expression in HMC3 cells. **g** Densitometric quantification of CD206 and CD86 expression (*n* = 6). **h** Gene set enrichment analysis (GSEA) showing downregulation of genes associated with the “cytokine–cytokine receptor interaction” pathway in SIRT2-knockdown HMC3 cells compared with control cells. **i** Representative transmission electron microscopy (TEM) images of LEVs and SEVs negatively stained with uranyl acetate. Scale bar: 200 nm. **j** Size distribution of LEVs and SEVs measured by TEM (*n* = 100–120 particles per group). **k** Western blot analysis of EVs-associated markers (Alix, Flotillin2, ARF6, and CD63) in LEVs and SEVs isolated from control and SIRT2-knockdown HMC3 cells. **l** Particle size distribution profiles of LEVs and SEVs determined by nanoparticle tracking analysis (NTA). **m** Representative fluorescence images showing the uptake of DiI-labeled LEVs and SEVs by HMC3, U87, and SH-SY5Y cells. Scale bar: 20 μm. **n** Quantification of DiI-labeled EVs uptake in HMC3, U87, and SH-SY5Y cells, indicating that HMC3 microglia internalize both LEVs and SEVs more efficiently than the other cell types. **o** Schematic illustration of the intranasal administration route and the proposed nose-to-brain delivery pathway of extracellular vesicles. **p** Immunofluorescence staining for Iba1⁺ microglia, GFAP⁺ astrocytes, and NeuN⁺ neurons in the olfactory bulb and hippocampus of mice after intranasal administration of DiI‑labeled LEVs or SEVs. CD63 co‑localization confirmed EVs internalization. DiI alone served as a negative control. Scale bars: 20 μm (overview) and 10 μm (insets). **q** Schematic overview of the experimental workflow for EVs isolation, characterization, and cellular uptake. Data are presented as mean ± SEM. *P < 0.05, **P < 0.01, ****P < 0.0001, ns: not significant

Stable SIRT2 knockdown (SIRT2-KD) HMC3 cell lines were generated by lentiviral transduction. Western blot analysis confirmed efficient knockdown, with SIRT2 protein levels reduced by approximately 80% compared with control cells (Fig. 1a–b). Previous reports have shown that SIRT2 knockdown promotes M2 polarization in BV2 microglial cells, a phenotype associated with anti-inflammatory functions [49]. To determine whether a similar shift occurs in HMC3 cells, we assessed the expression of CD86 (M1 marker) and CD206 (M2 marker) by immunofluorescence and Western blot. SIRT2-KD cells exhibited increased CD206 expression and decreased CD86 expression (Fig. 1c–g) relative to controls. Transcriptomic analysis further revealed downregulation of genes involved in the cytokine–cytokine receptor interaction pathway in SIRT2-KD cells (Fig. 1h), supporting a phenotypic transition toward an anti-inflammatory M2-like state.

To isolate EVs subtypes, LEVs and SEVs secreted by HMC3 cells were purified using differential ultracentrifugation. Vesicles derived from control and SIRT2-KD HMC3 cells were accordingly designated as LEVs-control, SEVs-control, LEVs-SIRT2-KD, and SEVs-SIRT2-KD. Transmission electron microscopy (TEM) revealed that both LEVs and SEVs displayed characteristic cup-shaped or semi-spherical morphologies with clear lipid bilayer membranes (Fig. 1i), and exhibited the expected size difference (Fig. 1j). Western blot analysis confirmed the enrichment of established EVs markers, including CD63, Alix, flotillin-2, and ARF6, in both populations (Fig. 1k). Nanoparticle tracking analysis (NTA) further substantiated their distinct size profiles: LEVs showed a broader diameter distribution, with mean sizes of 269.7 nm for LEVs-control and 288.6 nm for LEVs-SIRT2-KD, whereas SEVs were predominantly around 130 nm, with average diameters of 159.5 nm and 143.9 nm for SEVs-control and SEVs-SIRT2-KD, respectively (Fig. 1l). Importantly, SIRT2 knockdown did not significantly alter the yield or size distribution of either EVs subtype, as determined by NTA and protein quantification (Fig. S1).

We next assessed the capacity of these EVs to be taken up by neural cells, both in culture and after intranasal administration. In vitro, DiI-labeled LEVs and SEVs were internalized by human microglia (HMC3), astrocytes (U87), and neuronal cells (SH-SY5Y) within 24 h (Fig. 1m). Notably, microglia exhibited a significantly higher uptake efficiency than the other cell types under identical conditions (Fig. 1n), suggesting a preferential tropism. In vivo, following seven days of intranasal administration (Fig. 1o), DiI-labeled EVs were detected in microglia, astrocytes, and neurons within the olfactory bulb and hippocampus, with significant co‑localization with CD63 (Fig. 1p and Fig. S2), confirming that these particles can traverse the nasal pathway and reach the brain. To exclude non-specific labeling from free dye, mice receiving DiI alone served as a negative control. In these animals, only faint DiI fluorescence and sparse CD63-negative cellular uptake were observed, whereas EVs-treated groups showed robust CD63-positive signals (Fig. 1p). Collectively, these data demonstrate that intranasally administered EVs are efficiently internalized by neural cells in the brain, and their preferential uptake by microglia (Fig. 1q) suggests they may be harnessed to modulate glial responses in disease-relevant regions.

### LEVs-SIRT2-KD administered intranasally attenuate cognitive deficits in APP/PS1 mice

**Fig. 2:**
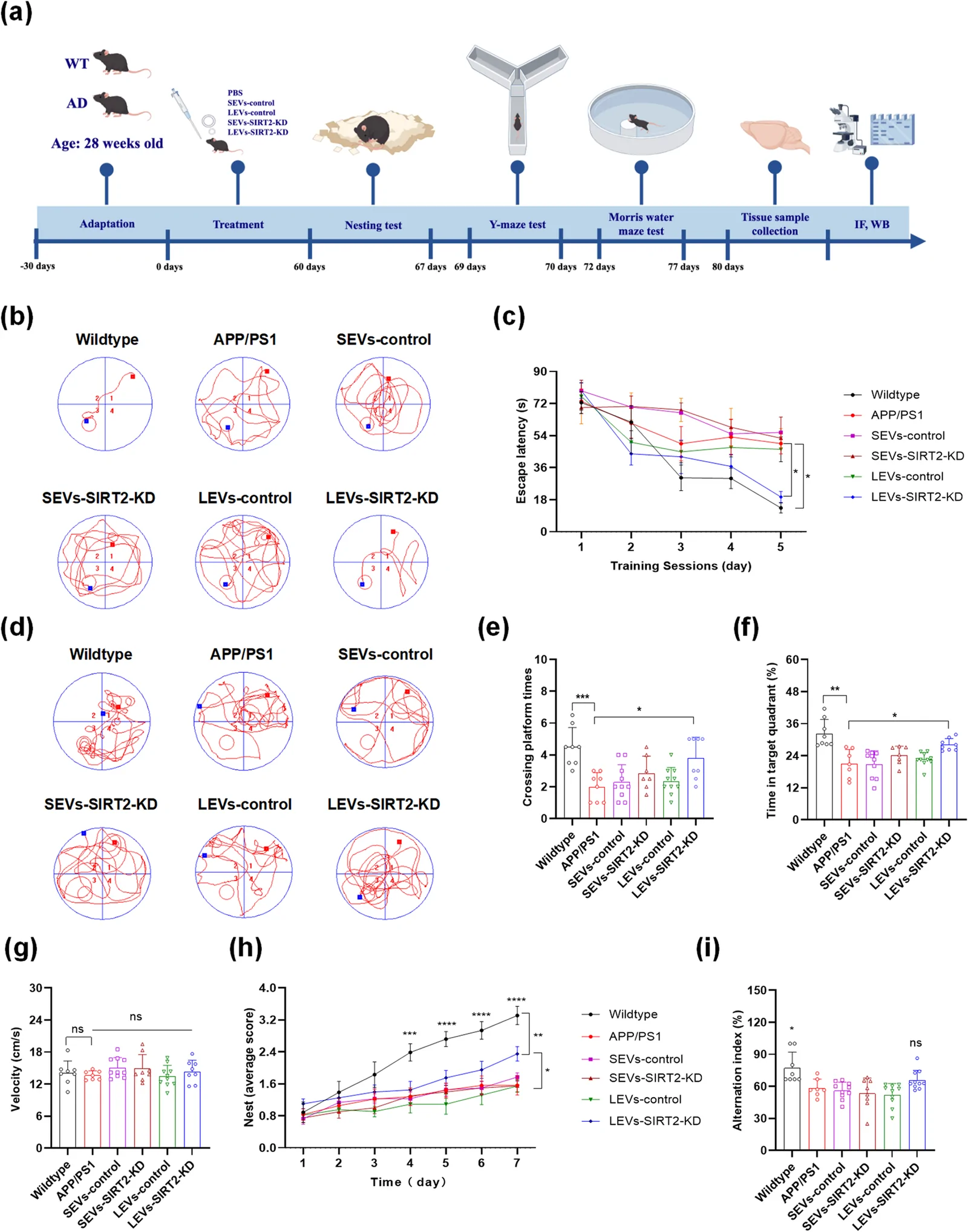
Intranasal administration of LEVs-SIRT2-KD ameliorates cognitive deficits in APP/PS1 transgenic mice. **a** Schematic illustration of the in vivo experimental timeline. **b** Representative swimming trajectories of mice during training sessions from day 1 to day 5. **c** Escape latency to locate the hidden platform across five consecutive training days. **d** Representative swim paths during the probe trial. **e** Number of platform crossings during the probe trial. **f** Time spent in the target quadrant during the probe trial. **g** Swimming speed across all experimental groups during the Morris water maze test. Wild-type: *n* = 8; APP/PS1: *n* = 7; SEVs-control: *n* = 10; LEVs-control: *n* = 10; SEVs-SIRT2-KD: *n* = 8; LEVs-SIRT2-KD: *n* = 8. **h** Nest-building scores assessed for each group. Wild-type: *n* = 9; APP/PS1: *n* = 9; SEVs-control: *n* = 11; LEVs-control: *n* = 11; SEVs-SIRT2-KD: *n* = 9; LEVs-SIRT2-KD: *n* = 9. **i** Spontaneous alternation index measured in the Y-maze test. Wild-type: *n* = 8; APP/PS1: *n* = 7; SEVs-control: *n* = 10; LEVs-control: *n* = 10; SEVs-SIRT2-KD: *n* = 8; LEVs-SIRT2-KD: *n* = 10. Given that our experimental design is not fully crossed—wild‑type mice received no EVs treatment, whereas APP/PS1 mice received various treatments—we adopted separate statistical approaches as outlined below. The genotype effect (wild‑type vs. APP/PS1) was assessed using an unpaired Student’s t‑test. The treatment effect among APP/PS1 mice (PBS, SEVs‑control, LEVs‑control, SEVs‑SIRT2‑KD, LEVs‑SIRT2‑KD) was evaluated using one‑way ANOVA followed by Dunnett’s T3 multiple comparisons test. For the learning curve across training days (Fig. 2c) and the nest‑building scores (Fig. 2h), two‑way ANOVA was used, followed by Tukey’s post‑hoc test. Data are presented as mean ± SEM. *P < 0.05, **P < 0.01, ***P < 0.001, ****P < 0.0001, ns: not significant

To evaluate the therapeutic potential of HMC3-derived EVs, eight-month-old APP/PS1 mice and their wild-type littermates were randomly assigned to six groups and received intranasal administration of PBS, control EVs, or SIRT2-KD EVs every other day for two months (Fig. 2a). The dosing interval was determined by tracking the brain distribution of DiI-labeled EVs following a single intranasal dose (0.5 × 10⁹ particles, ~ 10 µg). Fluorescent signals appeared in the olfactory bulb at 0.5 h, spread to the cortex and hippocampus by 2 h, and markedly diminished by 48 h (Fig. S3), supporting an every-other-day regimen to maintain sustained exposure.

After 30 doses, cognitive function was assessed using the Morris water maze, nest-building, and Y-maze tests. In the hidden-platform task, all mice showed reduced escape latencies over five training days. APP/PS1 mice exhibited significantly longer latencies than wild-type controls, indicating impaired spatial learning. Notably, this deficit was rescued by LEVs-SIRT2-KD treatment, whereas SEVs-control, LEVs-control, or SEVs-SIRT2-KD had no effect (Fig. 2b–c). During the probe trial, APP/PS1 mice crossed the former platform location less frequently and spent less time in the target quadrant—deficits that were again significantly ameliorated by LEVs-SIRT2-KD (Fig. 2d–f). Swimming speeds did not differ among groups (Fig. 2g), ruling out motor or motivational confounds. Together, these results demonstrate that LEVs-SIRT2-KD specifically restores spatial learning and memory in APP/PS1 mice.

Complementing these findings, the nest-building test revealed that APP/PS1 mice had markedly lower nesting scores, reflecting impairments in activities of daily living. Intranasal LEVs-SIRT2-KD significantly improved nesting behavior compared to all other EVs treatments (Fig. 2h, S4). In the Y-maze, APP/PS1 mice showed a reduced spontaneous alternation index, suggesting impaired working memory. LEVs-SIRT2-KD treatment trended toward improvement, though this did not reach statistical significance (Fig. 2i). Collectively, these behavioral data indicate that LEVs-SIRT2-KD, but not SEVs or control LEVs, effectively alleviates cognitive impairments in APP/PS1 mice.

### LEVs-SIRT2-KD restore synaptic integrity and reduce amyloid plaque burden in APP/PS1 mice

**Fig. 3:**
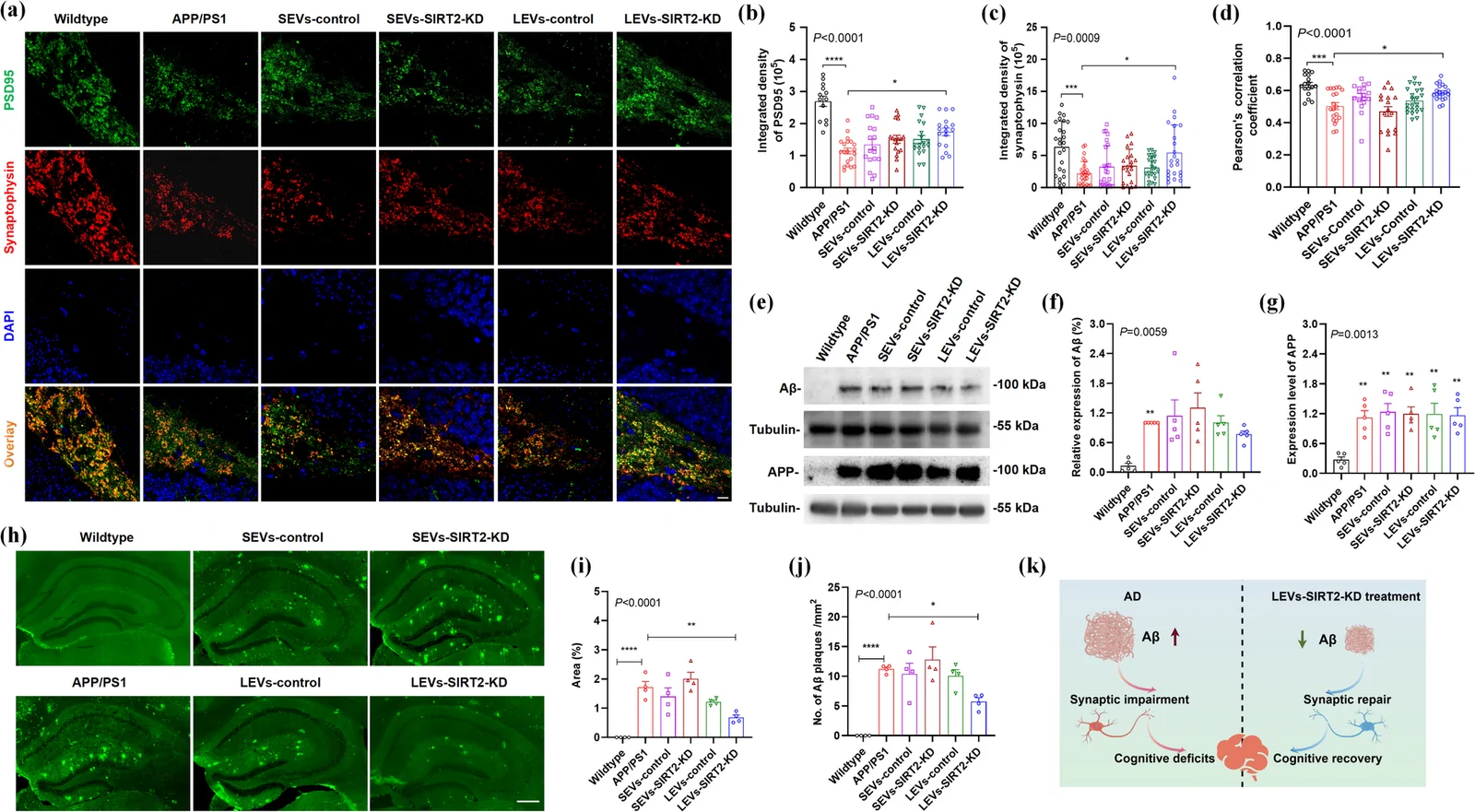
Intranasal administration of LEVs-SIRT2-KD reduces Aβ pathology and restores synaptic integrity in APP/PS1 mice. **a** Representative immunofluorescence images showing the distribution of PSD95 and SYN in the hippocampal region. Scale bar: 10 μm. **b-c **Quantitative analysis of PSD95 (**b**) and SYN (**c**) fluorescence intensity. **d** Pearson’s correlation coefficient for PSD95 and SYN co-localization. Data were obtained from 27 fields of view across three mice per group. **e** Western blot analysis of high-molecular-weight Aβ oligomers and amyloid precursor protein (APP) in hippocampal lysates. **f**-**g** Densitometric quantification of high-molecular-weight Aβ oligomers (**f**) and APP (**g**) protein levels (*n* = 5 per group). **h** Representative immunofluorescence staining of Aβ plaque deposition in the hippocampus. Scale bar: 200 μm. **i-j** Quantification of Aβ plaque area fraction (**i**) and density (**j**) in the hippocampus (*n* = 4 per group). **k** Schematic summary of the therapeutic effects of LEVs-SIRT2-KD treatment in APP/PS1 mice. Data are presented as mean ± SEM. *P < 0.05, **P < 0.01, ***P < 0.001, ****P < 0.0001

Given the profound cognitive improvements conferred by LEVs-SIRT2-KD, we next examined their impact on synaptic integrity and Aβ pathology. Immunofluorescence analysis of hippocampal sections revealed a marked reduction in the expression of the postsynaptic marker PSD95 and the presynaptic marker synaptophysin (SYN) in APP/PS1 mice compared to wild-type controls. Notably, treatment with LEVs-SIRT2-KD, but not other EVs, significantly restored the levels of both proteins and enhanced their synaptic co-localization (Fig. 3a–d), indicating improved synaptic density and integrity.

In parallel, consistent with the cognitive deficits, 10-month-old APP/PS1 mice exhibited extensive Aβ plaque deposition in the hippocampus. Intranasal administration of LEVs-SIRT2-KD robustly reduced both the number and total area of Aβ plaques, whereas control EVs or SEVs-SIRT2-KD had no significant effect (Fig. 3h–j). Of note, treatment with LEVs‑control or SEVs‑control resulted in modest, non‑significant reductions in Aβ plaque burden relative to PBS‑treated APP/PS1 mice, suggesting that control microglia‑derived EVs possess a degree of intrinsic bioactivity. However, a statistically significant decrease was observed only following LEVs‑SIRT2‑KD treatment, indicating that SIRT2 knockdown enriches LEVs with therapeutic cargo sufficient for functional efficacy. Western blot analysis revealed a trend toward decreased levels of high-molecular-weight Aβ oligomers in the LEVs-SIRT2-KD group, although this did not reach statistical significance. No significant changes in amyloid precursor protein (APP) expression were observed across groups (Fig. 3e–g). The dissociation between APP expression and Aβ pathology suggests that LEVs-SIRT2-KD may promote Aβ clearance rather than suppress its production.

Together, these findings demonstrate that LEVs-SIRT2-KD facilitate synaptic repair and enhance the clearance of aggregated Aβ species, providing a structural and molecular basis for the cognitive recovery observed in APP/PS1 mice (Fig. 3k).

### LEVs-SIRT2-KD promote microglial recruitment to Aβ plaques and enhance phagocytic clearance

**Fig. 4:**
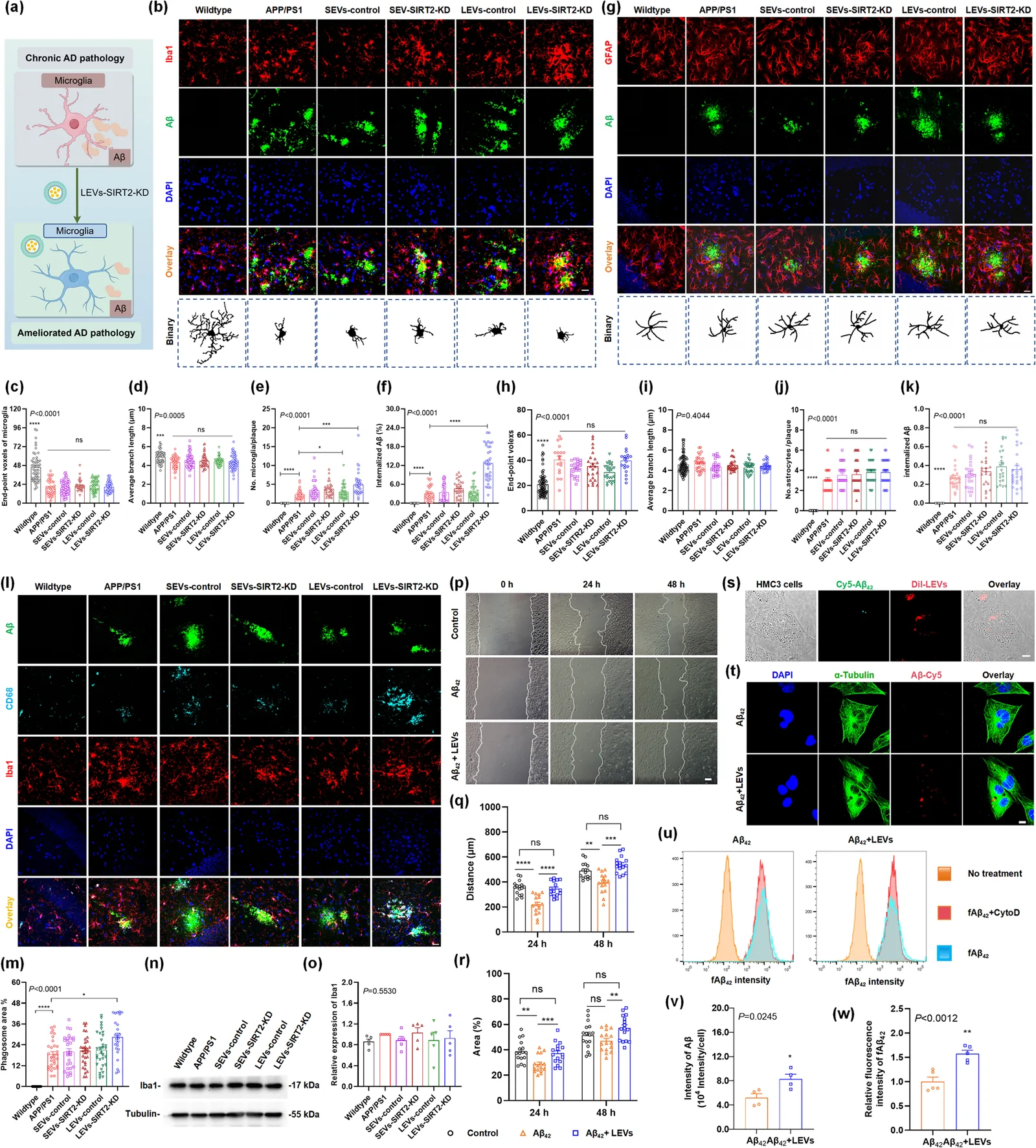
LEVs-SIRT2-KD enhance microglial recruitment to Aβ plaques and promote phagocytic activity. **a** Schematic illustration of the hypothesis tested in Fig. 4: whether the reduction in Aβ plaque burden achieved by LEVs‑SIRT2‑KD (quantified in Fig. 3h-j) is mediated through enhanced microglial recruitment and phagocytosis. **b** Representative immunofluorescence images showing Iba1⁺ microglia and Aβ plaques in the hippocampus, along with binary representations of microglial morphology. Scale bar: 20 μm. **c-f** Quantitative analysis of microglial endpoint voxels (**c**), mean branch length (**d**), cell number (**e**), and the proportion of microglia associated with Aβ internalization (**f**) around Aβ plaques (*n* = 36 fields from 4 mice per group). **g **Representative immunofluorescence images of GFAP⁺ astrocytes and Aβ plaques in the hippocampus, with binary representations of astrocytic morphology. Scale bar: 20 μm. **h-k** Quantitative analysis of astrocytic endpoint voxels (**h**), mean branch length (**i**), cell number (**j**), and the proportion of astrocytes associated with Aβ internalization (**k**) around Aβ plaques (*n* = 36 fields from 4 mice per group). No significant differences were detected between APP/PS1 mice and any EVs-treated group for these astrocytic parameters. **l** Immunofluorescence staining of Iba1, CD68, and Aβ in the hippocampus. Scale bar: 20 μm. **m** Quantification of the percentage of CD68⁺ phagosomes within Iba1⁺ microglia (*n* = 36 fields from 4 mice per group). **n-o** Western blot analysis (**n**) and densitometric quantification (**o**) of Iba1 protein levels in hippocampal lysates (*n* = 5 per group). **p-r** Migration assay of HMC3 cells following stimulation with Aβ_42_ oligomers. Representative images (**p**) and quantitative analysis of migration distance (**q**) and migration area (**r**) at 24 and 48 h (*n* = 16 per group). **s** Internalization of DiI‑labeled LEVs by HMC3 cells in the presence of Cy5‑Aβ₄₂. **t**,** v** Immunofluorescence analysis (**t**) and quantification (**v**) of Cy5-labeled Aβ_42_ oligomer phagocytosis by HMC3 cells (*n* = 4 per group). Scale bar: 20 μm. **u**,** w** Flow cytometric analysis of FAM-labeled Aβ_42_ oligomer uptake by HMC3 cells (**u**), with quantification of internalized fluorescence intensity after subtraction of CytoD-treated controls to exclude surface-bound signals (**w**) (*n* = 5 per group). Data are presented as mean ± SEM. *P < 0.05, **P < 0.01, ***P < 0.001, ****P < 0.0001

Having observed that LEVs-SIRT2-KD reduce Aβ plaque load without altering APP expression (Fig. 3), we hypothesized that these effects are mediated through enhanced Aβ clearance by glial cells, particularly microglia (Fig. 4a). Immunofluorescence analysis of hippocampal sections from 10-month-old APP/PS1 mice confirmed that microglia were recruited to the vicinity of Aβ plaques (Fig. 4b). These plaque-associated microglia displayed an amoeboid morphology, characterized by rounded cell bodies, reduced average branch length, and fewer process endpoints (Fig. 4c–d). In contrast, astrocytes surrounding the plaques exhibited an increase in process endpoints (Fig. 4g–i), suggesting a reactive phenotype.

Notably, continuous intranasal administration of LEVs-SIRT2-KD significantly increased the number of microglia surrounding Aβ plaques (Fig. 4b, e) and elevated the proportion of plaques partially or fully internalized by microglia (Fig. 4f). This effect was microglia-specific, as LEVs-SIRT2-KD did not alter astrocyte recruitment (Fig. 4g, j), Aβ uptake by astrocytes (Fig. 4k). Morphological parameters of both microglia and astrocytes remained unchanged following treatment (Fig. 4c–d, h–i). To further assess phagocytic activity, we co-stained for Iba1, CD68 (a phagolysosomal marker), and Aβ. In APP/PS1 mice, CD68⁺ phagosomes were sparse within microglia and showed limited co-localization with internalized Aβ. LEVs-SIRT2-KD treatment markedly increased both CD68 expression and the number of Aβ⁺ phagosomes in microglia (Fig. 4l–m), indicating enhanced phagocytic activity. Total Iba1 protein levels remained unchanged across groups (Fig. 4n–o). This is consistent with previous reports that microglial activation in AD primarily involves phenotypic changes of existing cells rather than global proliferation [50], and that microglial responses are spatially restricted to plaque vicinities [51].

These in vivo observations were corroborated by in vitro experiments. Scratch assays revealed that prolonged exposure to Aβ_42_ oligomers significantly suppressed the migration of HMC3 human microglial cells, whereas treatment with LEVs‑SIRT2‑KD markedly enhanced both migration distance and area (Fig. 4p–r). Consistently, immunofluorescence and flow cytometry analyses revealed that LEVs-SIRT2-KD substantially increased the uptake of fluorescently labeled Aβ₄₂ oligomers (Cy5-Aβ₄₂ and FAM-Aβ₄₂) by HMC3 cells (Fig. 4s–w). Of note, DiI-labeled LEVs were readily internalized by HMC3 cells in the presence of Cy5‑Aβ₄₂ (Fig. 4s) or FAM‑Aβ₄₂ (Fig. 6f), indicating that LEVs uptake occurs independently of Aβ oligomers.

Taken together, these findings demonstrate that LEVs-SIRT2-KD promote microglial recruitment to Aβ plaques and potentiate their phagocytic capacity, thereby facilitating the clearance of Aβ pathology in APP/PS1 mice (Fig. 4a).

### LEVs-SIRT2-KD enhance microglial energy metabolism by promoting glycolysis and oxidative phosphorylation

Having established that LEVs‑SIRT2‑KD enhance microglial recruitment and phagocytosis (Fig. 4), and given the high energy demands of these processes [16, 52], along with the metabolic dysfunction characteristic of AD microglia, we next asked whether these functional improvements are linked to changes in microglial energy metabolism. To address it, we examined the molecular landscape of the diseased brain and characterized the cargo of LEVs‑SIRT2‑KD by proteomic and acetylomic profiling, followed by functional metabolic assays.

**Fig. 5:**
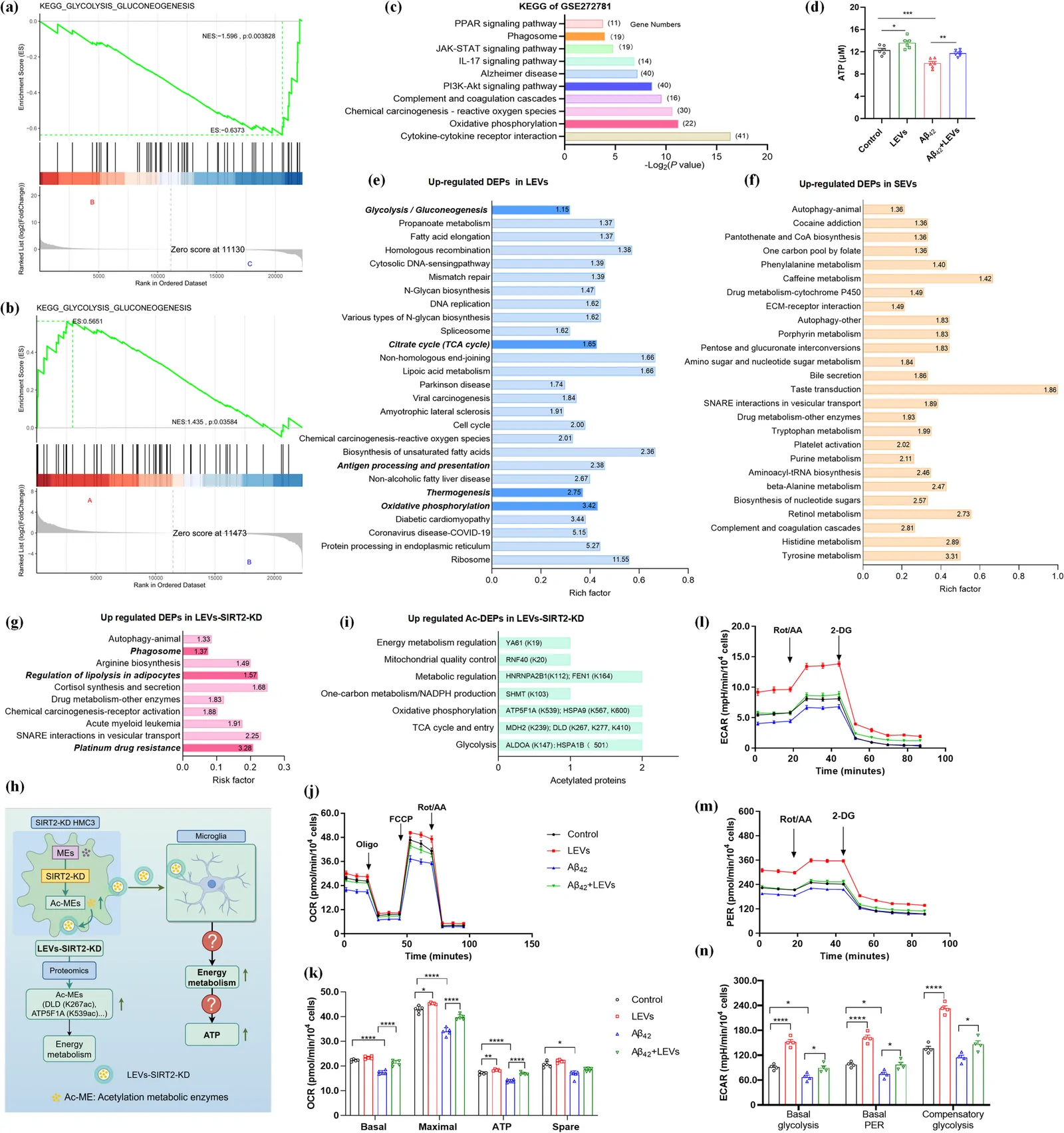
LEVs-SIRT2-KD enhance both glycolytic and oxidative phosphorylation activities in microglia. **a-b** Gene set enrichment analysis (GSEA) showing downregulation of glycolysis-related genes in the hippocampus of APP/PS1 mice compared with wild-type controls (**a**), and upregulation of these genes following LEVs-SIRT2-KD treatment compared with untreated APP/PS1 mice (**b**). **c** KEGG pathway enrichment analysis of differentially expressed genes in microglia from 9-month-old APP/PS1 mice versus age-matched wild-type mice (dataset GSE272781). **d** ATP production levels in HMC3 cells treated with Aβ_42_ oligomers in the presence or absence of LEVs-SIRT2-KD (*n* = 6). **e-g** KEGG enrichment analysis of upregulated differentially expressed proteins (DEPs) in LEVs-control versus SEVs-control (**e**), in SEVs-control versus LEVs-control (**f**), and in LEVs-SIRT2-KD versus LEVs-control (**g**). The values displayed at the end of each bar represent –log_10_ (*P* value). **h** Schematic illustration depicting that SIRT2 knockdown increases the levels of acetylated metabolic enzymes in HMC3 cells, which are subsequently packaged into LEVs and transferred to recipient cells to enhance energy metabolism. **i** KEGG enrichment analysis of upregulated differentially acetylated peptides in LEVs-SIRT2-KD compared with other EVs groups. **j** Oxygen consumption rate (OCR) analysis of HMC3 cells following 24 h treatment with Aβ_42_ oligomers and LEVs-SIRT2-KD. Sequential injections: oligomycin (1.5 µM), FCCP (1.5 µM), and rotenone/antimycin A (0.5 µM). **k** Quantitative assessment of basal respiration, maximal respiration, ATP production, and spare respiratory capacity derived from OCR measurements (*n* = 5). **l** Extracellular acidification rate (ECAR) analysis of HMC3 cells after 24 h treatment with Aβ_42_ oligomers and LEVs-SIRT2-KD, with 2-DG (50 mM) added as a glycolytic inhibitor. **m** Proton efflux rate (PER) analysis under the same experimental conditions. **n** Quantitative assessment of basal glycolysis, basal PER, and glycolytic reserve capacity derived from ECAR measurements (*n* = 4). Data are presented as mean ± SEM. *P < 0.05, **P < 0.01, ***P < 0.001, ****P < 0.0001

Transcriptomic profiling of whole hippocampal tissue revealed that glycolysis-related genes were significantly downregulated in APP/PS1 mice compared to wild-type controls (Fig. 5a). Notably, this glycolytic deficit was reversed by LEVs-SIRT2-KD treatment, which upregulated glycolysis-related gene expression in APP/PS1 hippocampus (Fig. 5b). In line with this, KEGG pathway analysis of differentially expressed genes from purified microglia of 9-month-old APP/PS1 mice (dataset GSE272781) showed significant enrichment of pathways related to inflammation (“cytokine-cytokine receptor interaction”), energy metabolism (“oxidative phosphorylation”), and signaling (“PI3K-Akt”) (Fig. 5c). These results indicate that microglia in the APP/PS1 brain exhibit profound metabolic disturbances, and suggest that LEVs‑SIRT2‑KD may exert their effects by modulating microglial energy metabolism.

We therefore investigated whether LEVs-SIRT2-KD carry molecular cargo capable of remodeling microglial metabolism. Quantitative proteomic profiling of all four EVs populations revealed striking baseline differences between the two EVs subtypes. LEVs-control were inherently enriched in proteins associated with energy metabolism, including oxidative phosphorylation, thermogenesis, and the citrate cycle (TCA cycle), alongside pathways linked to ribosome function and antigen processing and presentation (Fig. 5e). In contrast, SEVs-control were predominantly enriched in pathways involved in amino acid metabolism (tyrosine, histidine, phenylalanine), drug metabolism (cytochrome P450), autophagy, complement and coagulation cascades, and ECM-receptor interaction (Fig. 5f). Together, these distinct enrichment profiles indicates that LEVs are naturally equipped to support cellular energy production, protein synthesis, and immune modulation, whereas SEVs are more specialized in metabolic substrate processing and extracellular matrix interactions.

SIRT2 knockdown further remodeled LEV cargo. Compared with LEVs-control, LEVs-SIRT2-KD exhibited upregulated proteins involved in phagosome, autophagy, regulation of lipolysis in adipocytes, SNARE interactions in vesicular transport, and platinum drug resistance (Fig. 5g). While these pathways are not classical energy metabolism pathways, they are functionally intertwined with metabolic regulation: autophagy and lipolysis mobilize intracellular nutrients to sustain mitochondrial respiration [53, 54], SNARE-mediated trafficking supports phagosome maturation and receptor recycling [55, 56], and the platinum resistance pathway encompasses oxidative stress responses linked to mitochondrial function [57, 58]. Thus, this proteomic signature indicates that LEVs-SIRT2-KD acquire additional capacities for stress resilience, fuel mobilization, and vesicular trafficking.

Acetylomic analysis added a layer of mechanistic specificity. Among the 21 differentially acetylated peptides with increased abundance in LEVs-SIRT2-KD, we identified key metabolic enzymes, including DLDH (dihydrolipoamide dehydrogenase, a component of the pyruvate dehydrogenase complex), MDH2 (malate dehydrogenase), ATP5F1A (ATP synthase α subunit), ALDOA (aldolase A), and SHMT (serine hydroxymethyltransferase), along with regulatory proteins such as HNRNPA2B1 (implicated in glycolytic enzyme mRNA stability) and RNF40 (involved in mitophagy regulation) (Table 1). Although the limited number of differentially acetylated peptides precluded statistically significant KEGG pathway enrichment, the qualitative enrichment of energy metabolism-related proteins—with the TCA cycle, glycolysis, and oxidative phosphorylation categories containing the highest number of acetylated proteins—is notable (Fig. 5i).

Collectively, these multi-omics analyses suggest a model in which SIRT2 knockdown promotes the acetylation of key metabolic enzymes involved in glycolysis and oxidative phosphorylation within donor microglia, and these hyperacetylated enzymes are subsequently packaged into LEVs. Upon transfer to recipient microglia, they may enhance the activity of these metabolic pathways, thereby boosting cellular energy production (Fig. 5h).

**Table 1:** Enrichment of hyperacetylated mitochondrial metabolic proteins in LEVs following SIRT2 depletion

GeneName	Accession numbers	PTM Locations	A/B	*P* value	Subcellular localization	Function
HSPA9	P38646	(K600)	4.31	0.0023	Mitochondrial	Oxidative phosphorylation
SHMT	B4E1G2	(K103)	3.66	0.0180	Mitochondrial	One-carbon metabolism
HSPA9	P38646	(K567)	3.02	0.01329	Mitochondrial	Oxidative phosphorylation
HNRNPA2B1	P22626	(K112)	2.65	0.0022	Nuclear	Metabolic regulation
FEN1	B4DWZ4	(K164)	2.60	0.0035	Nuclear; Cytoplasmic	Metabolic regulation
ATP5F1A	P25705	(K539)	2.54	0.0328	Mitochondrial	Oxidative phosphorylation
DLDH	A0A024R713	(K277)	2.47	0.0290	Mitochondrial	TCA entry
DLDH	A0A024R713	(K267)	2.43	0.0127	Mitochondrial	TCA entry
DLDH	A0A024R713	(K410)	2.41	0.0361	Mitochondrial	TCA entry
HSPA1B	P0DMV9	(K501)	2.32	0.0019	Cytoplasmic	Chaperones
MDH	A0A024R4K3	(K239)	2.16	0.0286	Mitochondrial	TCA cycle
ALDOA	J3KPS3	(K147)	1.84	0.0064	Cytoplasmic	Glycolysis
RNF40	A0A0S2Z4Z2	(K20)	1.77	0.0361	Nuclear	Mitochondrial quality control
YA61	Q9NZ23	(K19)	NA	NA	Mitochondrial	Energy metabolism regulation

To directly test whether this cargo translates into functional metabolic enhancement, we performed Seahorse analysis to measure oxygen consumption rate (OCR) and extracellular acidification rate (ECAR) in HMC3 microglial cells. Aβ₄₂ exposure profoundly suppressed mitochondrial respiration, as evidenced by reduced basal respiration, maximal respiration, ATP-linked respiration, and spare respiratory capacity (Fig. 5j–k). Glycolytic function was similarly impaired, with significant decreases in extracellular acidification rate (ECAR), proton efflux rate (PER), basal glycolysis, and glycolytic reserve (Fig. 5l–n). Thus, Aβ₄₂ drives a broad metabolic failure in microglia, compromising both major ATP-generating pathways—a finding consistent with the pathway enrichment observed in APP/PS1 microglia (Fig. 5c). Remarkably, LEVs-SIRT2-KD treatment reversed this phenotype. In Aβ₄₂-exposed microglia, LEVs-SIRT2-KD restored OCR and ECAR to near-control levels across all measured parameters (Fig. 5j–n). Consistent with these findings, LEVs‑SIRT2‑KD also rescued Aβ₄₂‑induced ATP depletion (Fig. 5d).

Moreover, LEVs-SIRT2-KD alone—in the absence of Aβ_42_ stress—significantly elevated both OXPHOS and glycolysis in naive microglia, demonstrating that these vesicles actively potentiate energy metabolism rather than merely counteracting Aβ toxicity. Taken together, these findings establish that LEVs-SIRT2-KD, through their unique protein and acetylation cargo, concurrently enhance both glycolytic and oxidative phosphorylation pathways in recipient microglia, thereby restoring the metabolic fitness required for effective phagocytosis and migration.

### LEVs-SIRT2-KD enhance microglial migration and phagocytosis via metabolic restoration

**Fig. 6:**
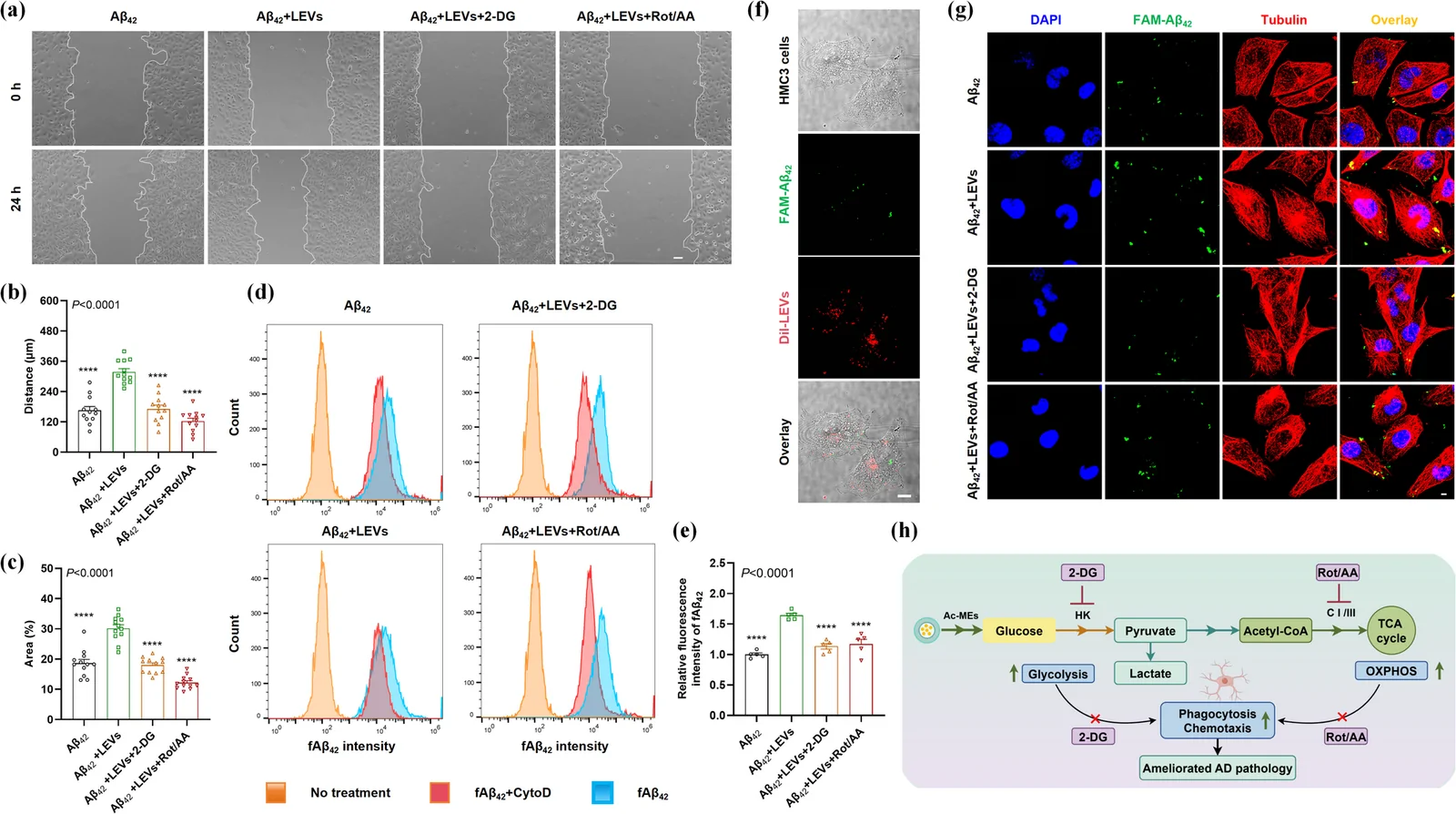
LEVs-SIRT2-KD enhance microglial migration and phagocytosis through modulation of mitochondrial energy metabolism.** a** Representative images of HMC3 cell migration following 24 h exposure to Aβ_42_ oligomers. **b-c** Quantitative analysis of migration distance (**b**) and migration area (**c**) in HMC3 cells after 24 h Aβ_42_ stimulation (*n* = 12 per group). **d** Flow cytometric analysis of FAM-Aβ_42_ oligomer phagocytosis by HMC3 cells after 24 h exposure (1 µM), with rotenone/antimycin A (Rot/AA, 0.25 µM) to inhibit mitochondrial complexes I and III, and 2-deoxy-D-glucose (2-DG, 30 mM) to inhibit glycolysis. **e** Quantification of FAM-Aβ_42_ phagocytosis based on flow cytometry data. **f** Internalization of DiI‑labeled LEVs by HMC3 cells in the presence of FAM‑Aβ₄₂. **g** Immunofluorescence assessment of HMC3 cell phagocytosis of FAM-labeled Aβ_42_ oligomers. Scale bar: 20 μm. **h** Schematic diagram illustrating the distinct inhibitory mechanisms of Rot/AA and 2-DG on oxidative phosphorylation (OXPHOS) and glycolysis, respectively, alongside the proposed regulatory role of LEVs-SIRT2-KD in enhancing microglial energy metabolism. Data are presented as mean ± SEM. *P < 0.05, **P < 0.01, ***P < 0.001, ****P < 0.0001

The preceding experiments established that LEVs-SIRT2-KD concurrently enhance both OXPHOS and glycolysis in microglia. To determine whether this metabolic restoration is causally linked to the observed improvements in microglial function, we performed pharmacological inhibition experiments. HMC3 microglial cells were treated with LEVs-SIRT2-KD in the presence or absence of rotenone/antimycin A (Rot/AA, inhibitors of mitochondrial complexes I and III) to block OXPHOS, or 2-deoxy-D-glucose (2-DG) to inhibit glycolysis. Scratch assays revealed that the enhanced migration induced by LEVs-SIRT2-KD was nearly abolished by either Rot/AA or 2-DG, with significant reductions in both migration distance and area (Fig. 6a–c). Similarly, the increased phagocytic uptake of fluorescently labeled Aβ₄₂ oligomers (FAM-Aβ₄₂) following LEVs-SIRT2-KD treatment was markedly attenuated by both metabolic inhibitors, as quantified by immunofluorescence and flow cytometry (Fig. 6d–g). Notably, inhibition of either pathway alone was sufficient to abrogate the functional benefits conferred by LEVs-SIRT2-KD, indicating that both OXPHOS and glycolysis are essential for the enhanced microglial migration and phagocytosis.

These results provide direct causal evidence that LEVs-SIRT2-KD improve microglial motility and Aβ clearance by upregulating mitochondrial energy metabolism. The dependence on both ATP-generating pathways underscores the critical role of metabolic fitness in supporting the energy-demanding processes of cytoskeletal rearrangement and phagosome formation that underlie effective microglial function (Fig. 6h) [16, 52].

## Discussion

In this study, we demonstrate that LEVs derived from SIRT2-knockdown HMC3 cells effectively alleviate AD pathology and cognitive deficits in APP/PS1 mice by remodeling the energy metabolism of recipient microglia. Our findings yield three key insights: (a) LEVs, rather than SEVs, are identified as the primary mediators of the therapeutic benefits of SIRT2 silencing; (b) enhanced microglial OXPHOS and glycolysis are causally linked to the restoration of phagocytic and migratory functions; and (c) intranasally delivered, microglia-derived LEVs represent a promising cell-free therapeutic strategy for neuroimmune metabolic modulation in AD. Mechanistically, multi-omics profiling revealed a cohesive molecular picture underlying this therapeutic effect: LEVs naturally possess an inherent predisposition for energy metabolism, SIRT2 knockdown equips them with additional stress-resilience and catabolic machinery, and specific acetylation marks on key metabolic enzymes provide a direct mechanistic link to enhanced enzymatic activity in recipient cells.

The functional divergence between LEVs and SEVs observed in our study is particularly noteworthy. While both subtypes share a common tropism for microglia in vitro, only LEVs derived from SIRT2-KD microglia (LEVs-SIRT2-KD) significantly improved cognition and reduced Aβ pathology. This dichotomy likely stems from their distinct biogenesis and molecular cargo. According to the MISEV2018 guidelines, LEVs are generated via outward budding of the plasma membrane [59], and their molecular cargo is expected to reflect the identity and physiological state of the parent cell [60]. Consistent with this, our proteomic data reveal that LEVs-control are naturally enriched in energy metabolism and protein synthesis pathways, whereas SEVs-control are enriched in amino acid metabolism, drug metabolism, and autophagy. This intrinsic specialization suggests that LEVs are inherently poised to deliver metabolic signals.

SIRT2 knockdown further amplified this specialization. LEVs-SIRT2-KD acquired additional cargo involved in phagosome function, vesicular trafficking, and stress-resilience pathways (autophagy, lipolysis, platinum drug resistance). Although not classical metabolic pathways, these processes converge on energy metabolism by mobilizing intracellular nutrients, supporting vesicular delivery of metabolic enzymes, and buffering oxidative stress—thereby creating a permissive environment for sustained energy production.

Acetylomic analysis provided a direct mechanistic link. Among the 21 hyperacetylated peptides in LEVs-SIRT2-KD, we identified key metabolic enzymes spanning glycolysis (ALDOA), TCA cycle entry and flux (DLDH, MDH2), oxidative phosphorylation (ATP5F1A), and one-carbon metabolism (SHMT), along with regulatory proteins such as HNRNPA2B1 and RNF40. Although the limited number of acetylated peptides precluded statistical significance, the qualitative enrichment of acetylation marks of the core energy metabolism pathways aligns with the functional enhancement of both glycolysis and oxidative phosphorylation in recipient microglia. Collectively, these multi-omics analyses reveal that LEVs-SIRT2-KD combine an inherent metabolic predisposition, stress-resilience machinery, and precise acetylation marks on key enzymes—a molecular payload that positions them to reprogram recipient microglial metabolism, a capacity not shared by other EVs.

A central and unexpected finding is that LEVs-SIRT2-KD concurrently enhance both OXPHOS and glycolysis in microglia. This dual enhancement appears, at first glance, to challenge the classical immunometabolic paradigm, which posits that pro-inflammatory (M1) microglia rely on glycolysis, whereas anti-inflammatory (M2) microglia favor OXPHOS [61]. However, our data point to a more nuanced interpretation. In the context of AD, microglia are subjected to chronic metabolic stress induced by Aβ, leading to a broad impairment of cellular energy metabolism that compromises both glycolytic and oxidative phosphorylation pathways, ultimately resulting in reduced ATP production [18]. This notion is supported by our Seahorse analyses, which demonstrate that exposure to Aβ_42_ oligomers suppresses both OXPHOS and glycolysis in HMC3 cells, effectively pushing them into a state of metabolic failure. Therefore, rather than driving microglia toward a specific polarized phenotype, LEVs-SIRT2-KD appear to function as “metabolic rehabilitators.” By simultaneously potentiating both major ATP-generating pathways, they restore metabolic flexibility and re-establish overall energy homeostasis. This metabolic enhancement provides the necessary fuel for cytoskeletal rearrangements that underpin enhanced microglial migration and phagocytosis—two highly energy-demanding processes that are markedly impaired within the AD microenvironment [16, 17, 52]. The abrogation of the functional benefits conferred by LEVs-SIRT2-KD upon pharmacological inhibition of OXPHOS and glycolysis provides compelling causal evidence supporting this mechanistic model.

These mechanistic insights not only elucidate how LEVs-SIRT2-KD enhance microglial function but also provide a strong rationale for their translational potential. From a clinical application perspective, our study highlights the potential of combining EVs technology with a non-invasive intranasal route. The efficient delivery of LEVs-SIRT2-KD to the olfactory bulb and hippocampus, coupled with their uptake by microglia, offers several advantages over existing strategies. Unlike systemically administered small molecule SIRT2 inhibitors, which suffer from poor BBB penetration and off-target peripheral effects [33], intranasal LEVs provide a targeted, brain-centric delivery with minimal systemic exposure. Furthermore, compared to viral vector-based gene therapy, our approach utilizes acellular vesicles derived from a stable, well-characterized human cell line, potentially mitigating concerns over insertional mutagenesis and uncontrolled immunogenicity [34]. The absence of overt toxicity over two months of repeated administration, while preliminary, supports the feasibility of this approach for chronic diseases like AD.

Several limitations of this study warrant consideration and point to future directions. First, our multi-omics analyses have identified a unique cargo associated with LEVs-SIRT2-KD, but which specific component(s) among these candidates directly mediate the metabolic reprogramming remains unknown. Future work must move beyond bulk proteomics to functionally validate the responsible protein(s) or acetylated modification(s). Second, despite adherence to MISEV guidelines [59], our LEVs preparation remains inherently heterogeneous and contains minor SEVs contamination; it is therefore more accurately described as a large EVs-enriched fraction. Future application of single-vesicle analysis technologies will be essential to determine whether the observed therapeutic activity originates from a specific LEVs subpopulation or reflects a collective effect of the heterogeneous vesicle population [62]. Finally, while no acute toxicity was observed, a comprehensive evaluation of the long-term effects of chronic intranasal EVs administration on nasal mucosa integrity, systemic immunity, and peripheral organ function is essential prior to clinical translation.

## Conclusions

**Fig. 7:**
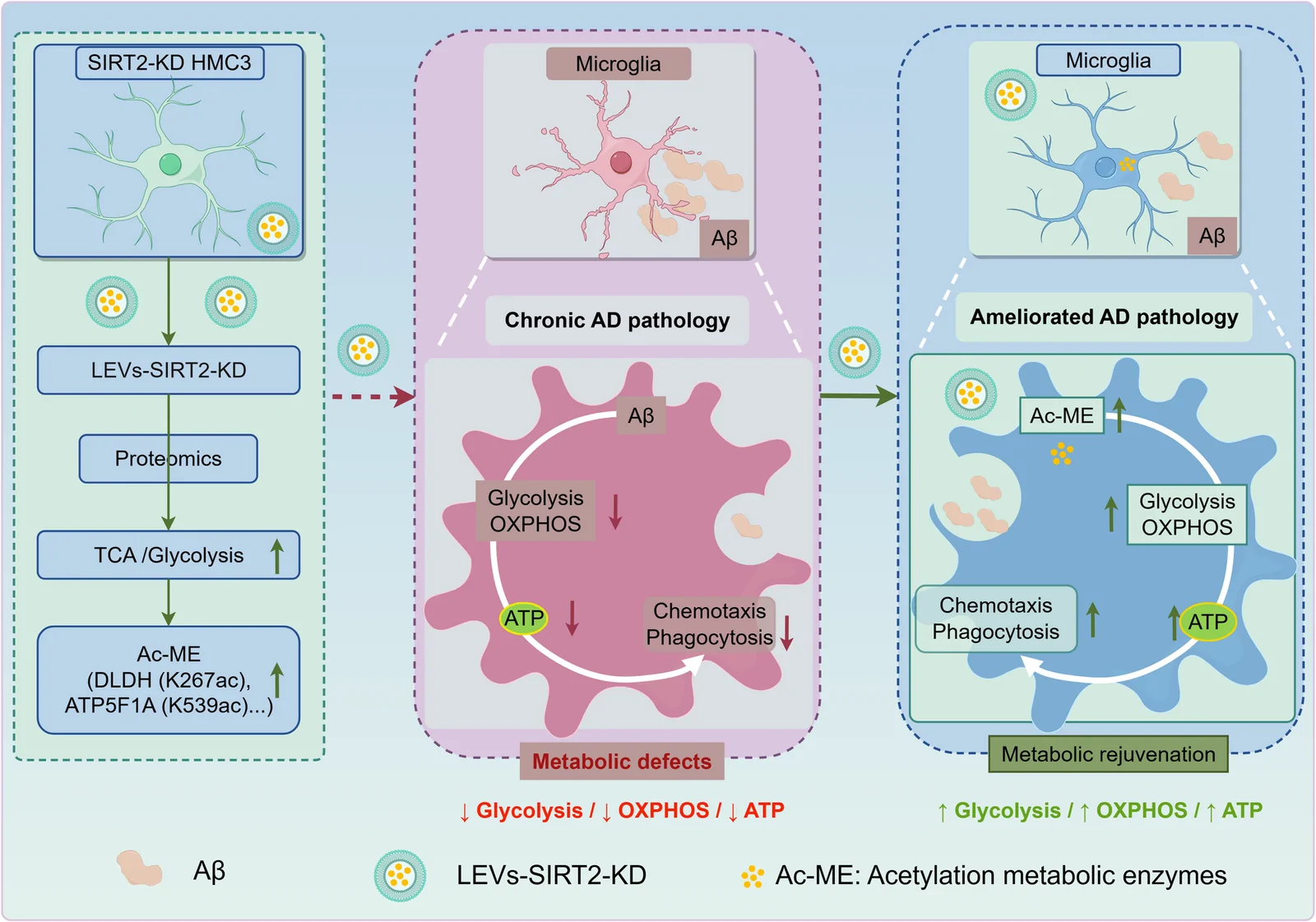
Schematic model of the therapeutic mechanism of LEVs‑SIRT2‑KD in APP/PS1 mice. LEVs derived from SIRT2‑knockdown HMC3 microglia carry hyperacetylated metabolic enzymes (Ac‑ME), including DLDH (K267ac), ATP5F1A (K539ac), and others. Once delivered to recipient microglia, these LEVs enhance both glycolysis and oxidative phosphorylation (OXPHOS)—a process we refer to as metabolic rejuvenation—leading to increased ATP production. This metabolic recovery enables microglial chemotaxis toward Aβ plaques and phagocytic clearance, thereby reducing plaque burden and ameliorating AD pathology. Ac‑ME: acetylation‑modified metabolic enzymes

In conclusion, this study demonstrates that LEVs from SIRT2-knockdown microglia transfer a unique metabolic cargo that reprograms recipient microglial energy metabolism. This metabolic rejuvenation restores their capacity to migrate toward and clear Aβ plaques, ultimately rescuing synaptic and cognitive deficits in an AD mouse model (Fig. 7). By uncovering LEVs as the key functional vesicle subtype and establishing a causal link between metabolic restoration and microglial function, our findings not only advance the understanding of SIRT2 biology in neuroinflammation but also provide a strong rationale for developing EVs-based neuroimmune metabolic therapies for AD and potentially other neurodegenerative disorders.

## Supplementary Information

Supplementary Material 1.

Supplementary Material 2.

## Data Availability

No datasets were generated or analysed during the current study.

## Abbreviations

AD: Alzheimer’s disease
Aβ: Amyloid-beta
APP: Amyloid precursor protein
ARIA: Amyloid-related imaging abnormalities
BBB: Blood-brain barrier
BCA: Bicinchoninic acid
DAPI: 4’,6-diamidino-2-phenylindole
DEPs: Differentially expressed proteins
DIA: Data-independent acquisition
DMSO: Dimethyl sulfoxide
ECAR: Extracellular acidification rate
EVs: Extracellular vesicles
FAM: Fluorescein amidite
FDR: False discovery rate
GO: Gene ontology
GSEA: Gene set enrichment analysis
HCD: Higher-energy collisional dissociation
HPLC: High-performance liquid chromatography
Iba1: Ionized calcium-binding adapter molecule 1
iRT: Internal retention time
KD: Knockdown
KEGG: Kyoto Encyclopedia of Genes and Genomes
LEVs: Large extracellular vesicles
M/M2: Microglia polarization states (pro-inflammatory / anti-inflammatory)
MEM: Minimum essential medium
MISEV: Minimal Information for Studies of Extracellular Vesicles
MS: Mass spectrometry
NTA: Nanoparticle tracking analysis
OCR: Oxygen consumption rate
OXPHOS: Oxidative phosphorylation
PBS: Phosphate-buffered saline
PER: Proton efflux rate
PFA: Paraformaldehyde
PMSF: Phenylmethylsulfonyl fluoride
PSD95: Postsynaptic density protein 95
PVDF: Polyvinylidene fluoride
QC: Quality control
RIPA: Radioimmunoprecipitation assay
Rot/AA: Rotenone and antimycin A
SDS-PAGE: Sodium dodecyl sulfate–polyacrylamide gel electrophoresis
SEM: Standard error of the mean
SEVs: Small extracellular vesicles
SIRT2: Sirtuin 2
SYN: Synaptophysin
TCA cycle: Tricarboxylic acid cycle
TEM: Transmission electron microscopy
2-DG: 2-deoxy-D-glucose
