# Supplementary material for: Metabolic reprogramming via SIRT2-deficient microglial large extracellular vesicles ameliorates alzheimer’s pathology
Source: J Neuroinflammation. 2026 Jul 17;23:340. doi: 10.1186/s12974-026-03956-3 (PMC13660121; doi:10.1186/s12974-026-03956-3)
Supplement: Supplementary file 1 [file 12974_2026_3956_MOESM2_ESM.pdf]

Fig. 1a

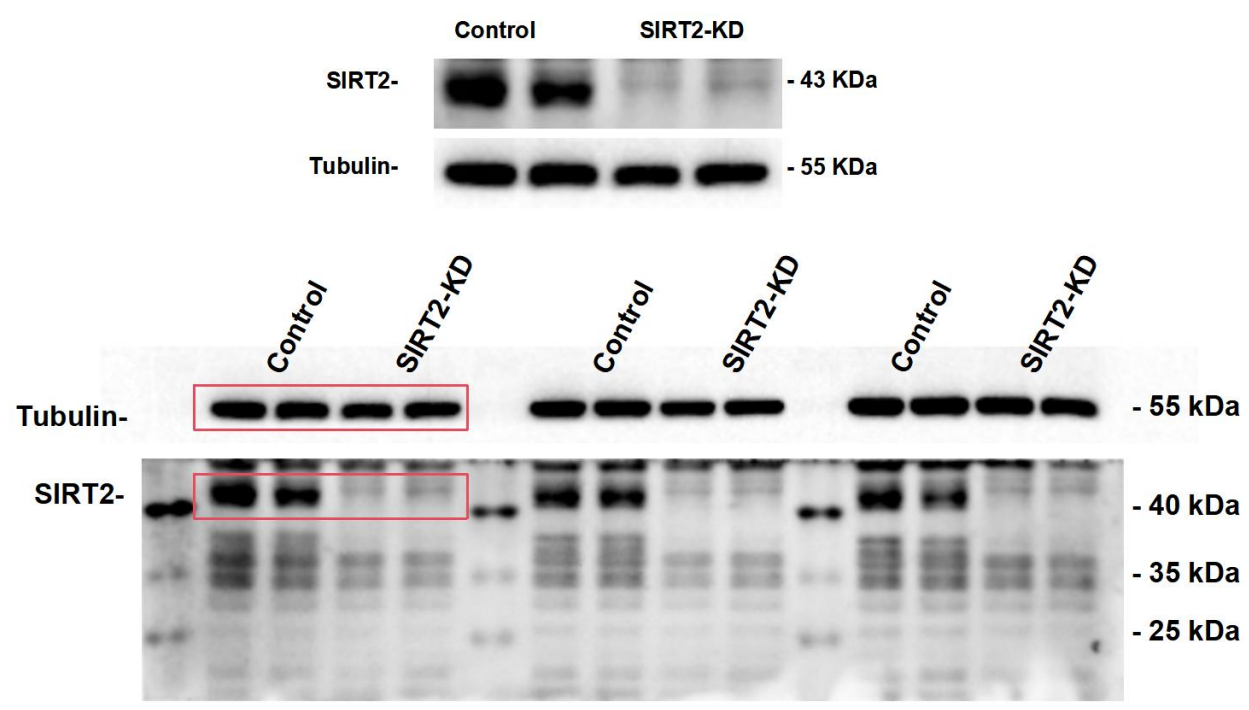

Fig. 1i

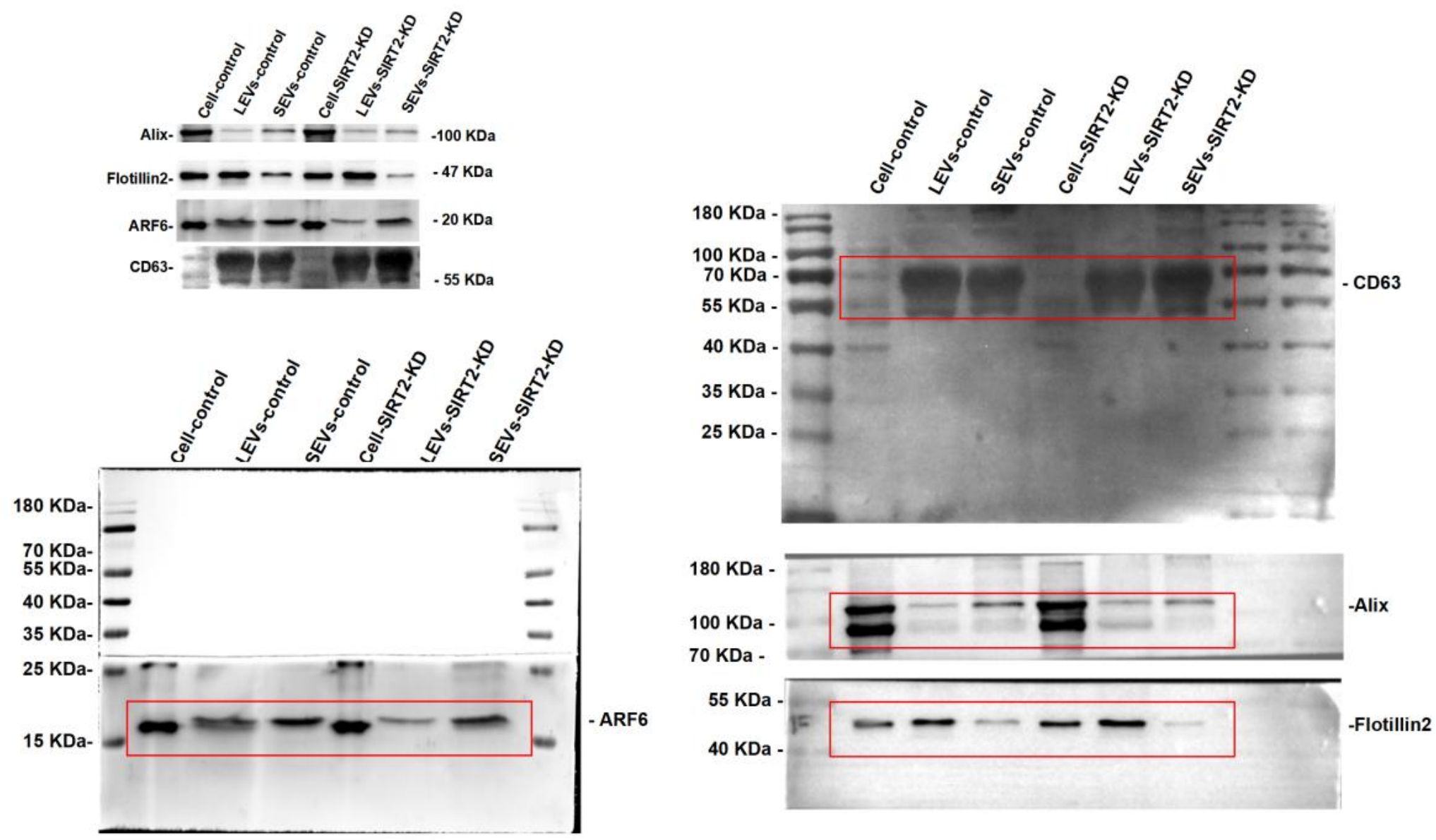

Fig. 3e

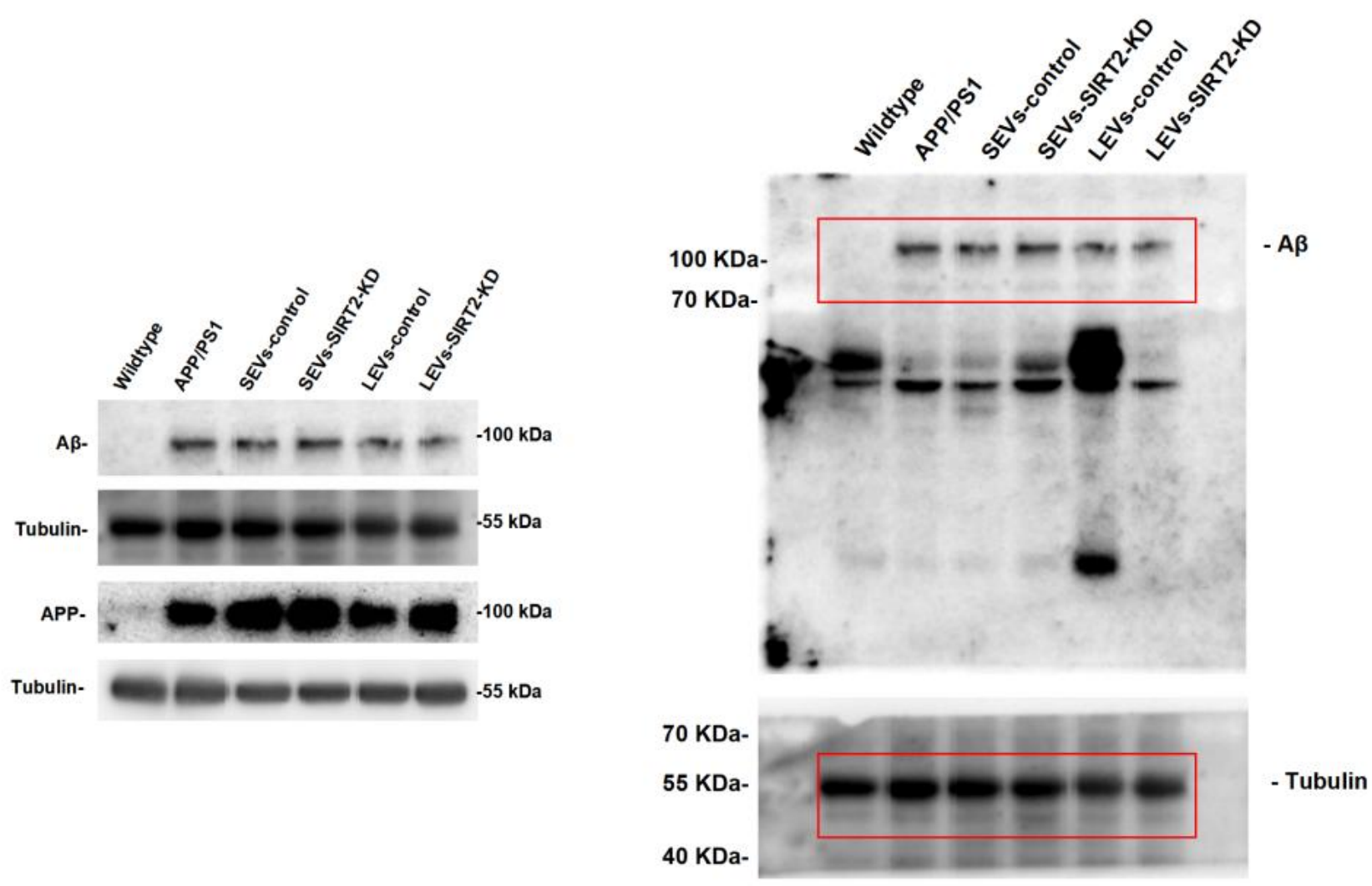

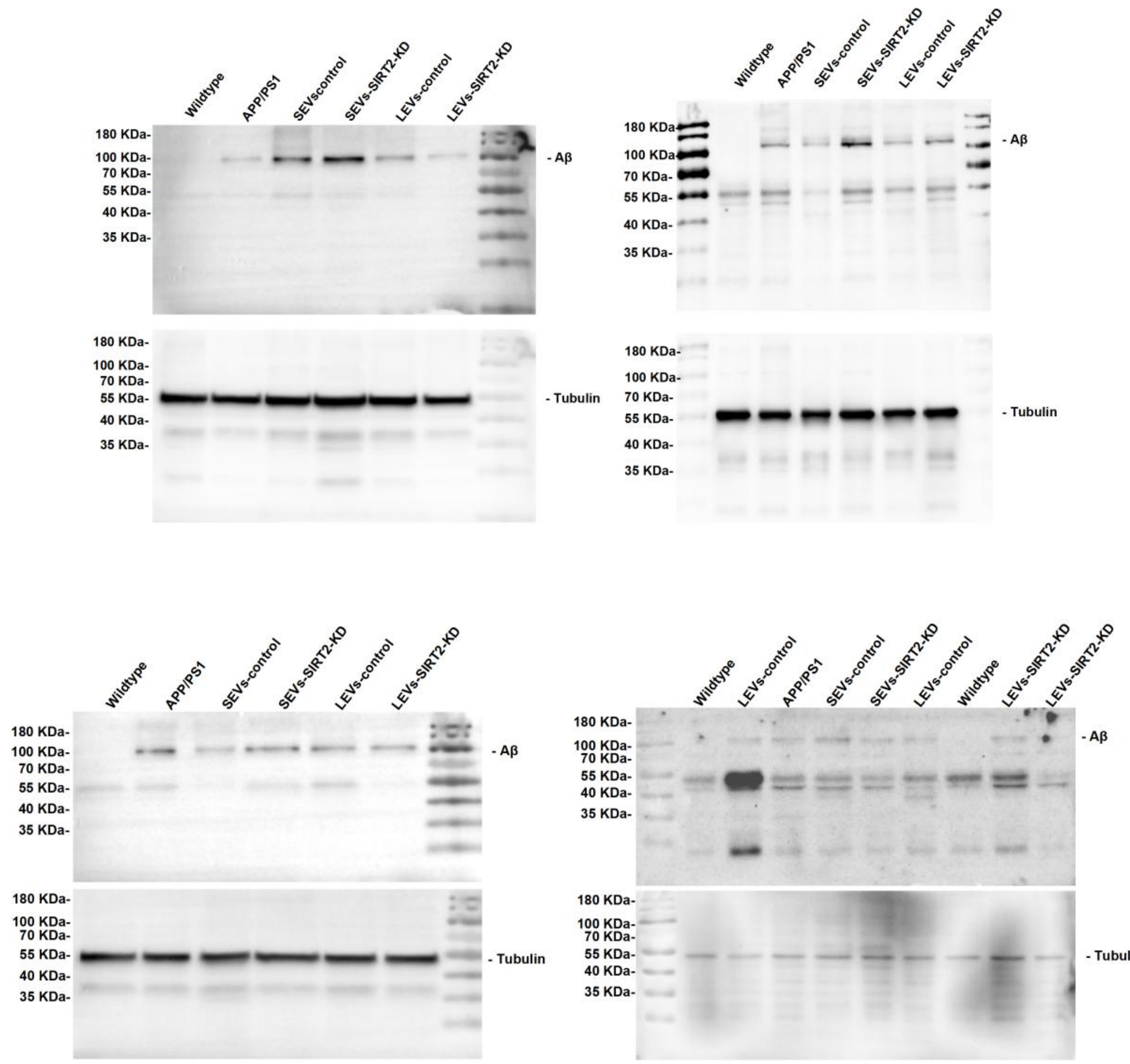
Wildtype
APP/PS1
SEVs-control
SEVs-SIRT2-KD
LEVs-control
LEVs-SIRT2-KD
180 KDa-
100 KDa-
70 KDa-
55 KDa-
40 KDa-
35 KDa-
- Aβ
- Tubulin

Fig. 3e

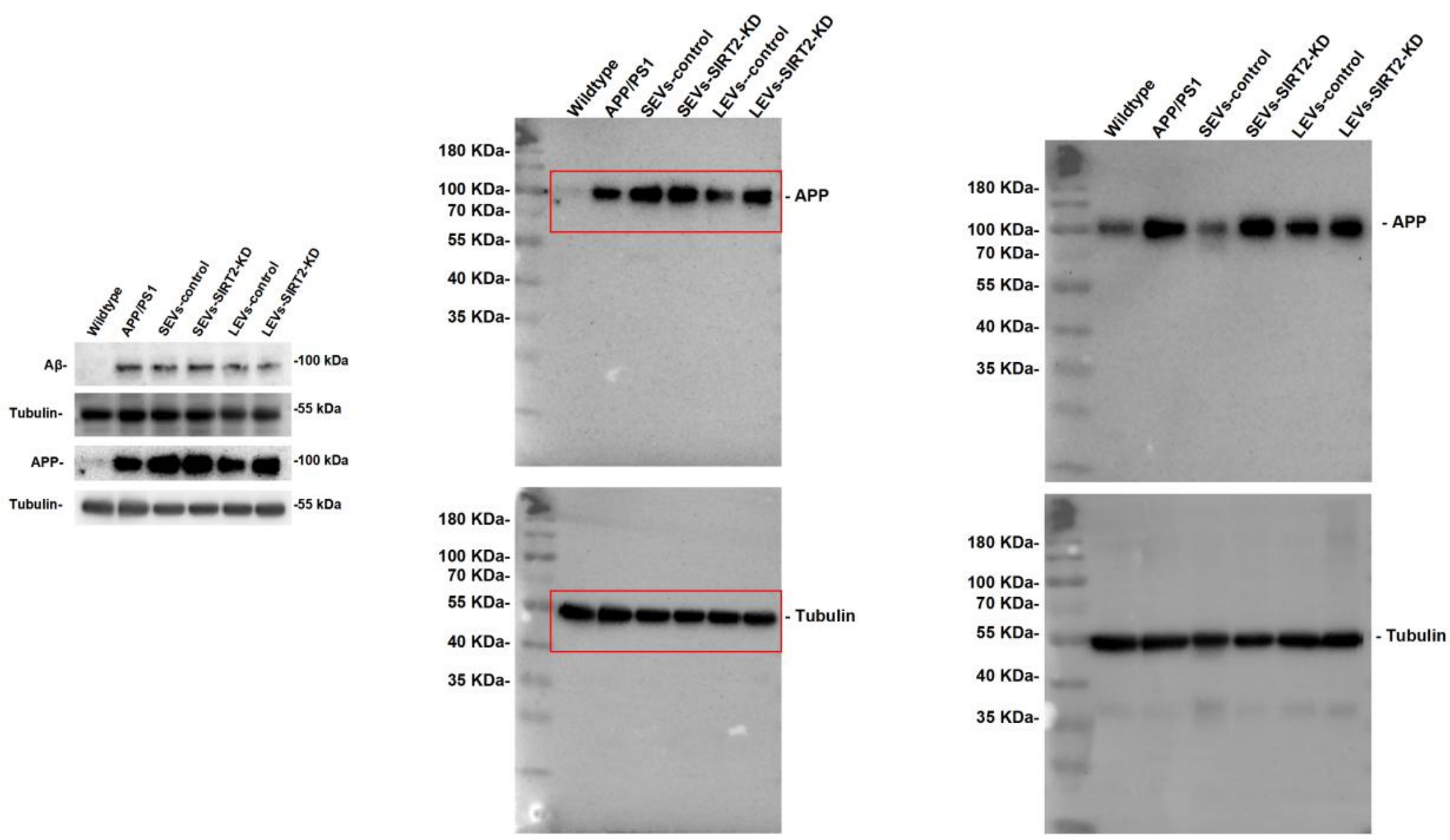
Wildtype
APP/PS1
SEVs-control
SEVs-SIRT2-KD
LEVs-control
LEVs-SIRT2-KD
Aβ-
Tubulin-
APP-
-100 kDa
-55 kDa
180 KDa-
100 KDa-
70 KDa-
55 KDa-
40 KDa-
35 KDa-
- APP
- Tubulin

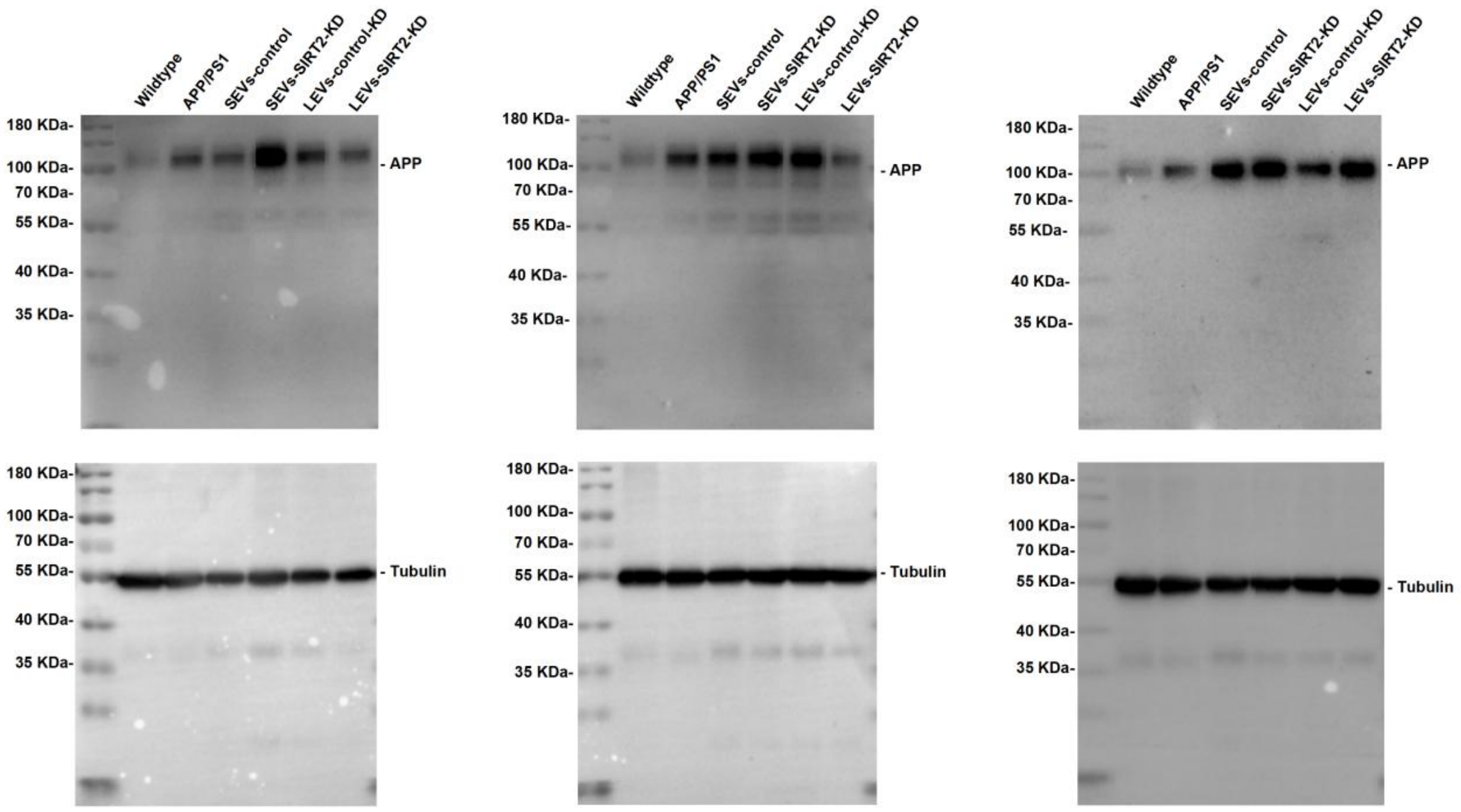
Wildtype
APP/PS1
SEVs-control
SEVs-SIRT2-KD
LEVs-control-KD
LEVs-SIRT2-KD
180 KDa-
100 KDa-
70 KDa-
55 KDa-
40 KDa-
35 KDa-
- APP
- Tubulin

Fig. 4n

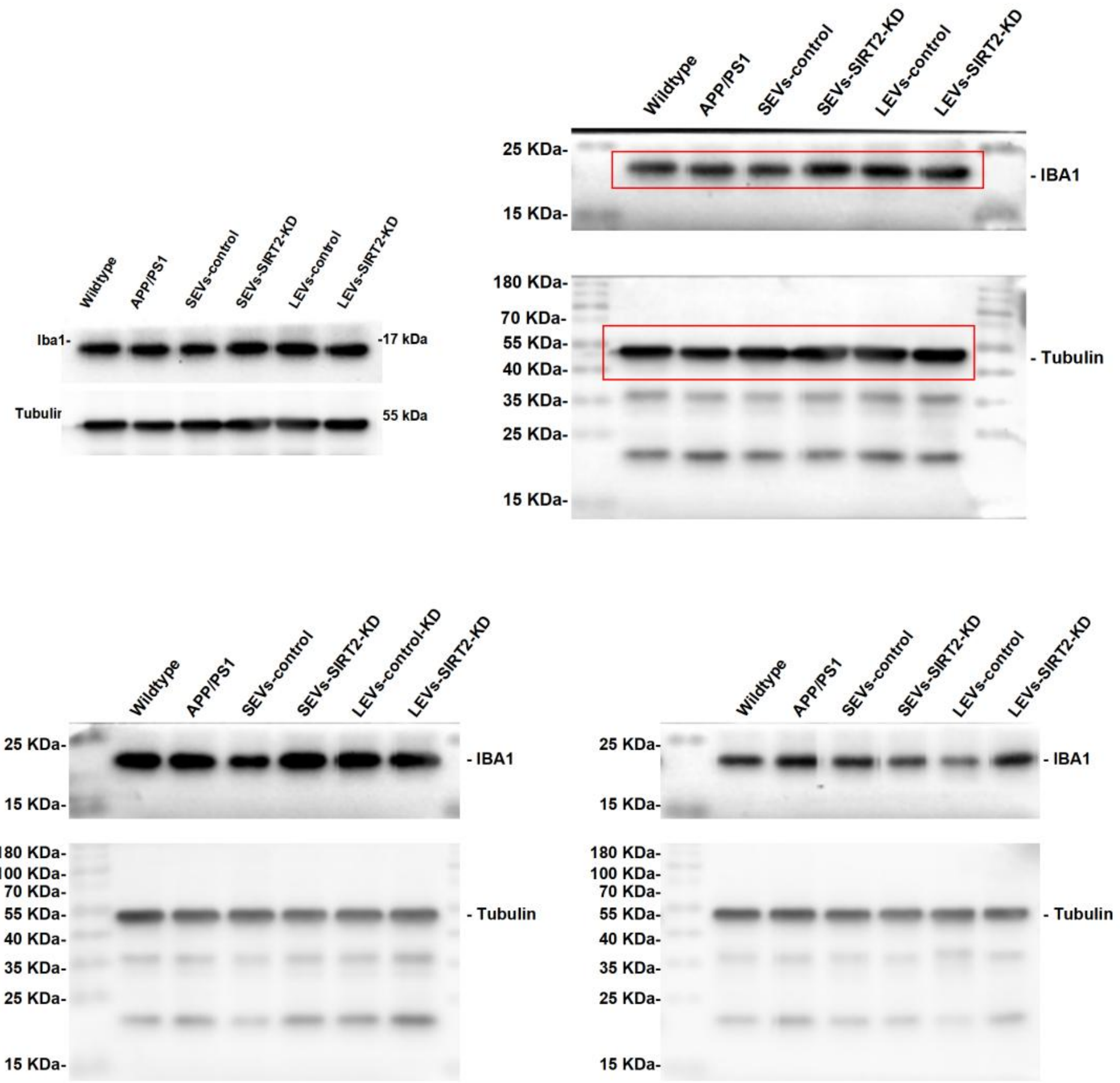
Wildtype
APP/PS1
SEVs-control
SEVs-SIRT2-KD
LEVs-control
LEVs-control-KD
LEVs-SIRT2-KD
Iba1-
-17 kDa
Tubulin
55 kDa
25 KDa-
15 KDa-
- IBA1
180 KDa-
100 KDa-
70 KDa-
55 KDa-
40 KDa-
35 KDa-
- Tubulin

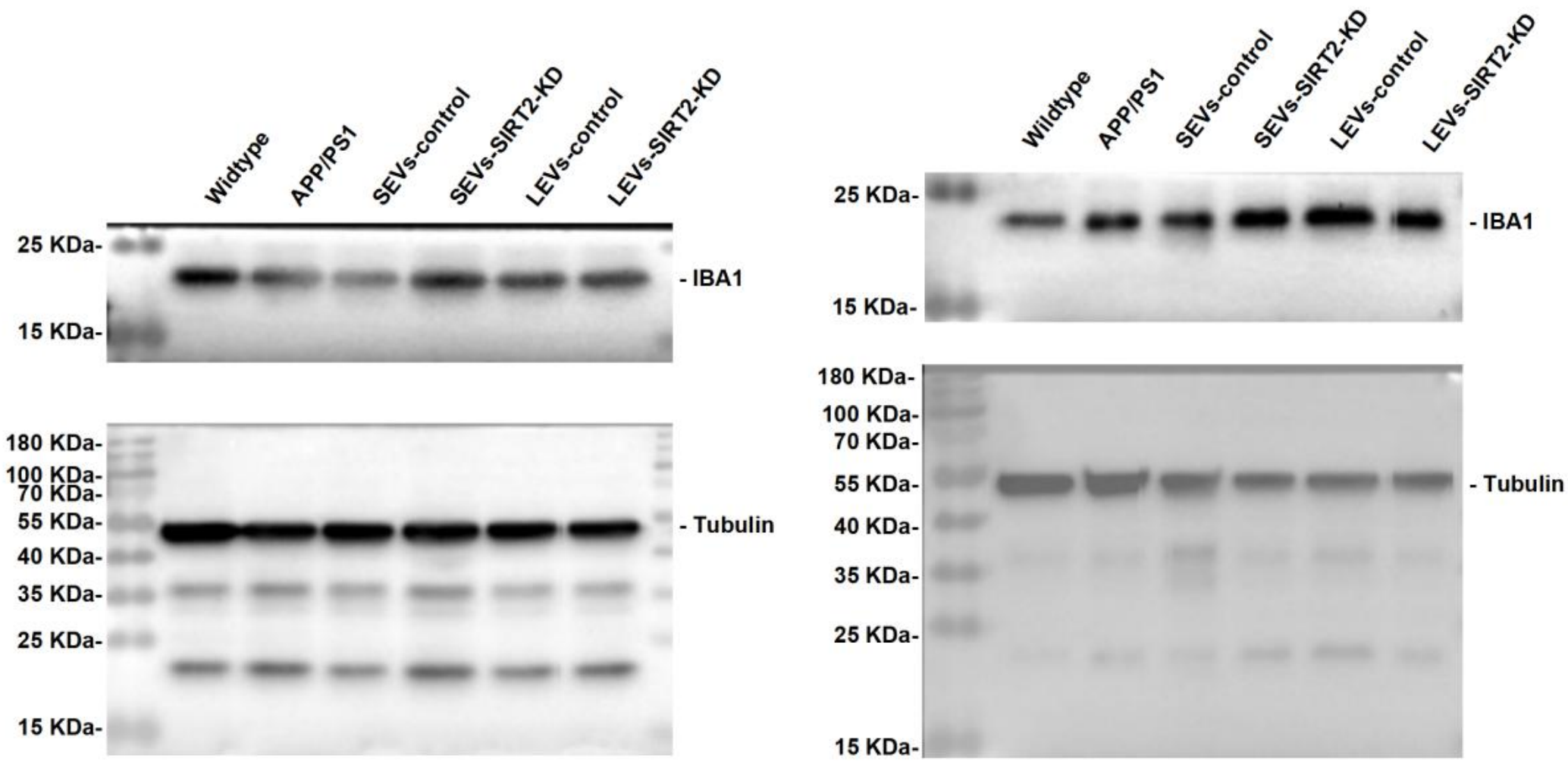
Widtype
APP/PS1
SEVs-control
SEVs-SIRT2-KD
LEVs-control
LEVs-SIRT2-KD
25 KDa-
15 KDa-
- IBA1
180 KDa-
100 KDa-
70 KDa-
55 KDa-
40 KDa-
35 KDa-
25 KDa-
15 KDa-
- Tubulin

Fig. 1f

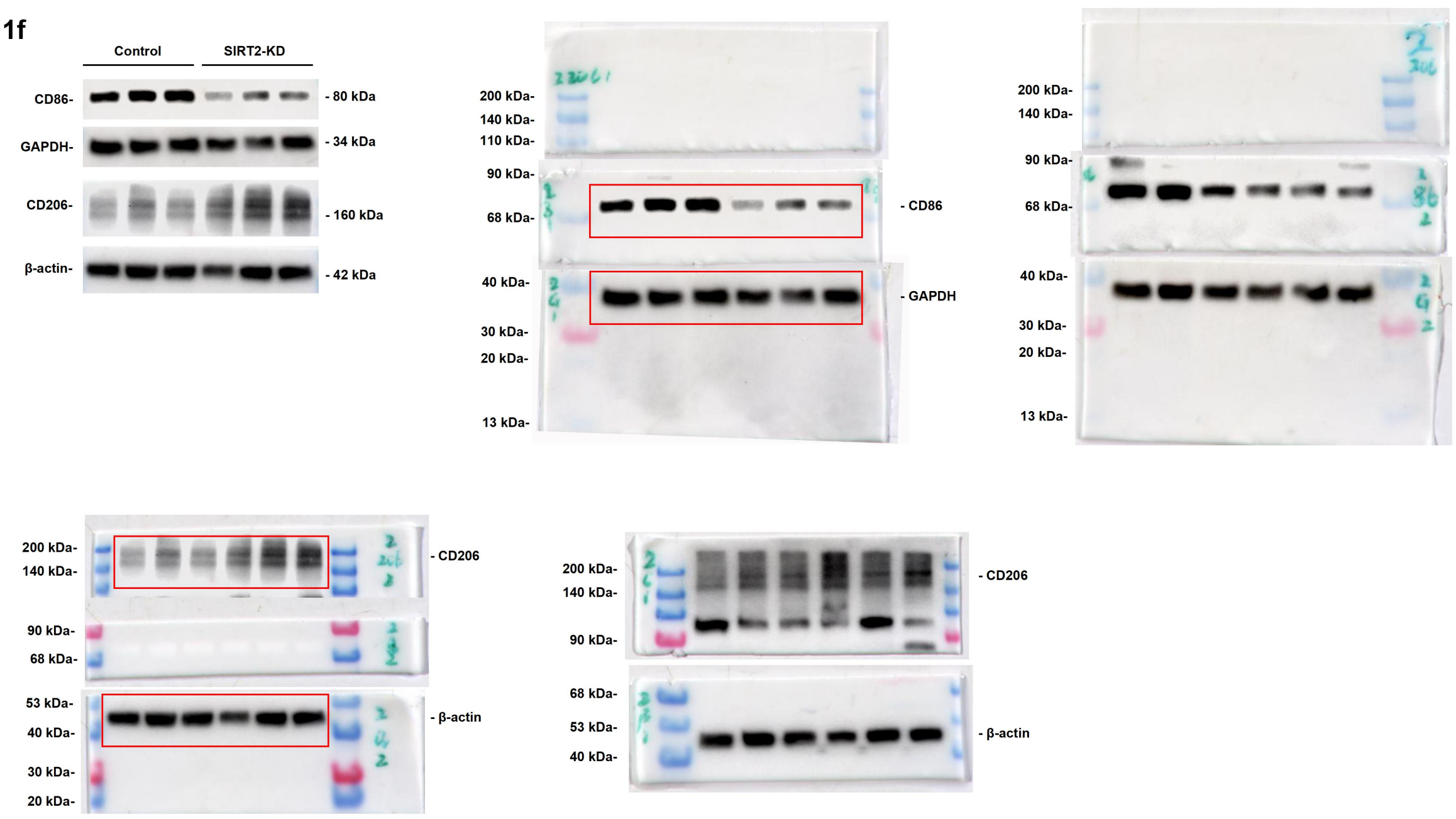
Control
SIRT2-KD
CD86-
- 80 kDa
GAPDH-
- 34 kDa
CD206-
- 160 kDa
β-actin-
- 42 kDa
200 kDa-
140 kDa-
110 kDa-
90 kDa-
68 kDa-
40 kDa-
30 kDa-
20 kDa-
13 kDa-
- CD86
- GAPDH
53 kDa-
- CD206
- β-actin
