# Supplementary material for: Metabolic reprogramming via SIRT2-deficient microglial large extracellular vesicles ameliorates alzheimer’s pathology
Source: J Neuroinflammation. 2026 Jul 17;23:340. doi: 10.1186/s12974-026-03956-3 (PMC13660121; doi:10.1186/s12974-026-03956-3)
Supplement: Supplementary file 2 [file 12974_2026_3956_MOESM1_ESM.docx]

**Fig. S1** SIRT2 knockdown in HMC3 cells does not affect EV secretion. ****(a–b)**** Nanoparticle tracking analysis (NTA) quantification of LEV concentration ****(a)**** and total particle number ****(b)**** secreted by HMC3 cells (1.6×10^6^ cells). ****(c–d)**** Bicinchoninic acid (BCA) protein assay quantification of LEV protein concentration ****(c)**** and total protein content ****(d)****. ****(e–f)**** NTA quantification of SEV concentration ****(e)**** and total particle number ****(f)****. ****(g–h)**** BCA quantification of SEV protein concentration ****(g)**** and total protein content ****(h)****. Data are presented as mean ± SEM. n = 6 per group, ns, not significant.

**Fig. S2** Immunofluorescence staining for Iba1⁺ microglia and CD63 (EVs marker) in the olfactory bulb (OB) and hippocampus (HIP) of mice after intranasal delivery of DiI‑labeled LEVs or SEVs. Scale bars: 10 μm.

**Fig. S3 Time-dependent distribution of LEVs in the mouse brain following intranasal administration.** OB: olfactory bulb; Hip: hippocampus; CX: cortex. Scale bar: 200 μm (left two columns) and 50 μm (right two columns, magnified views of the corresponding regions). The representative data shown are from three independent mice.

**Fig. S4** Representative images of the nest-building test.
